# Five-year mental health outcomes for children and young people presenting to GPs in England with psychiatric symptoms

**DOI:** 10.1016/S2215-0366(24)00038-5

**Published:** 2024-04-01

**Authors:** Morwenna Senior, Matthias Pierce, Vicky P Taxiarchi, Shruti Garg, Dawn Edge, Tamsin Newlove-Delgado, Sharon A.S. Neufeld, Kathryn M. Abel

**Affiliations:** 1 Centre for Women’s Mental Health, Faculty of Biology, Medicine and Health Sciences, University of Manchester, Manchester, UK; 2 Division of Neuroscience, School of Biological Sciences, University of Manchester, Manchester Academic Health Science Centre, Manchester, UK; 3 Division of Psychology & Mental Health, Faculty of Biology, Medicine & Health, The University of Manchester, Manchester, UK; 4 Equality, Diversity & Inclusion Research Unit, Greater Manchester Mental Health NHS Trust, Manchester, UK; 5 NIHR Manchester Biomedical Research Centre, Manchester, UK; 6 NHIR Greater Manchester Patient Safety Research Collaboration, Manchester, UK; 7 Medical School, University of Exeter, St Lukes Campus, Exeter, UK; 8 Department of Psychiatry, University of Cambridge, Cambridge, UK; 9 Greater Manchester Mental Health NHS Foundation Trust, Manchester, UK

## Abstract

**Background:**

Little information is available about what happens to children and young people (CYP) who attend GPs with psychological symptoms. Given significantly growing presentations, we examined five-year service-use in English primary care for CYP with neurodevelopmental or mental health symptoms/diagnoses.

**Methods:**

A retrospective cohort study of primary care (CPRD-Aurum database) identified 369,340 CYP (3-18 years) presenting between 2000-2016. Group-Based-Multi-Trajectory Models (GBMTM) identified clusters with similar 5-year trajectories of: mental health-related GP contacts, psychotropic prescriptions and specialist mental health contacts. Survival analysis subsequently examined associations between trajectory membership and self-harm hospitalisation or suicide.

**Results:**

In the best-fitting, seven group GBMTM, over a 5 year period, the largest group (‘low contact’, 51·2%) had no, or minimal additional service contact or psychotropic prescriptions. 13·0% were in a *moderate, non-pharmacological contact* group and 8·7% were in a *declining contact* group. Two groups had escalating contact (*year-5 escalating contact* - 6·9% and *year-4 escalating contact* - 5·2%). There were two *prolonged contact* groups (*prolonged GP contact -* 8·6%, and *prolonged specialist service/care contact -* 6·5%). Non-white ethnicity and presentation in earlier years (2000–2004) was associated with *low contact* group membership. The *prolonged specialist contact* group had highest risk of self-harm hospitalisation (Hazard Ratio vs. low-contact group 2·19, 95% CI 2·03 - 2·36) and suicide (HR 2·67 95% CI 1·72 - 4·14).

**Conclusions:**

Most CYP presenting to primary care with psychiatric symptoms/diagnoses have low or declining rates of ongoing contact. If these trajectories reflect symptomatic improvement, these findings provide reassurance for CYP and their caregivers; these trajectories may, however, reflect unmet need for some.

## Introduction

The number of children and young people (CYP) presenting to healthcare services with psychiatric symptoms (incorporating mental health and neurodevelopmental conditions) has been increasing over the last 20 years across many countries.^1^ For example, in England, between 2003 and 2018 increasing numbers presented to GPs with symptoms of anxiety, depression, autism spectrum conditions, ADHD, and self-harm;^2^ while there was a 108% increase in the number of CAMHS referrals in England between November 2017 and November 2022.^3^ It is likely that increased service use is driven by a combination of greater clinical need (the rate of 7 to 16 year olds with ‘probable mental disorder’ in survey data increased from 12·1% in 2017 to 18·0% in 2022), alongside an increase in help-seeking behaviour (37% of these surveyed CYP sought help from a medical professional in 2004 compared to 50% in 2022).^4^

As increasing numbers of children and young people present to services with psychiatric symptoms, important gaps in our understanding have emerged about what is likely to happen to them after they make contact. Previous research has tended to describe the trajectories of defined or discrete symptom clusters or diagnoses e.g. children with internalising and externalising symptoms,^5–7^ depressive symptoms,^8,9^ ADHD symptoms,^10^ and autistic traits.^11^ These support the notion that outcomes are heterogeneous: different symptom clusters are found to have distinct, developmentally sensitive trajectories; common difficulties frequently co-occur;^10^ and stable high, or increasing trajectories of common symptoms may be associated with greater risk of later mental health problems and poor educational attainment.^9^ However, previous reports may not generalise to the current population of CYP attending primary care because presentations may cross discrete diagnostic constructs and a complex interplay of clinical and non-clinical CYP, caregiver and healthcare system characteristics are likely to have determined when, how and why CYP present.

Where single diagnostic criteria are met, cohort studies can highlight associations between mental health problems in childhood and risk of disorder in adulthood.^12^ However, whilst important, these associations can overshadow evidence that prognosis is often good. For example, a meta-analysis of controls in randomised trials reported recovery rates for common mental disorder in CYP of 50% within one year,^13^ and trajectory studies of internalising, externalising and depressive symptoms have consistently identified latent groups with symptoms limited to childhood or adolescence.^6,8^

Describing average changes in CYP mental health outcomes can conceal heterogeneity in the course of these outcomes. Group-based trajectory analysis can address this limitation, and has been used to investigate CYP symptom trajectories;^10,14^ however, data on trajectories of help-seeking in real-world cohorts of CYP is lacking. Understanding trajectories in mental healthcare utilisation could help families understand what is most likely to happen to their children, elucidate care pathways and inform service planning.

We aimed to investigate service use outcomes for CYP in England five years after presentation to primary care with symptoms of a neurodevelopmental or mental health condition using primary care records. First, we describe the proportion of CYP experiencing different outcomes (further GP contact, prescription of a psychotropic drug and referral into/contact with specialist mental health services) for each of the five years following presentation to primary care with a psychiatric symptom. Second, we use group-based trajectory models to identify distinct trajectories groups of these outcomes for CYP presenting to primary care. Third, we examined whether baseline characteristics, expected to influence prognosis, treatment decisions or healthcare utilisation, were associated with trajectory group membership. Finally, we examined the clinical validity of the trajectory groups by assessing whether membership is associated with future indicators of severe psychiatric distress (hospitalisations for self-harm, death by suicide). These analyses were descriptive in nature, since service use outcomes for CYP presenting to primary care with psychiatric conditions have not previously been investigated.

## Methods

### Study design and participants

This retrospective cohort study uses anonymised primary care health records: the Clinical Practice Research Datalink Aurum database (CPRD-Aurum^15^) containing demographics, GP-recorded diagnoses and symptoms, GP-issued prescriptions and referrals to specialist healthcare. In June 2021, 25% of England’s population were registered with a participating practice. The cohort was linked to the Hospital Episode Statistics admitted patient care dataset (HES-APC) and the Office for National Statistics (ONS) mortality registry.

Our sample consisted of CYP aged 3-18 years presenting to primary care in England with a mental health, behavioural, or neurodevelopmental symptom between 1^st^ January 2000 and 9^th^ May 2016 (appendix p2 [flowchart]). This age range includes early neurodevelopmental presentations, and reflects criteria for child and adolescent mental health services in the UK. Each patient’s ‘eligibility period’ ranged from the latest date of: 3^rd^ birthday, registration at participating practice, or 1^st^ January 2000; until the earliest date of: day before 19^th^ birthday, death, transfer out of practice, last data collection from practice, 9^th^ May 2016. Within this period, patients were included if they had a recorded mental health, behavioural, or neurodevelopmental symptom or diagnosis, had acceptable data quality (appendix p3), and were eligible for linkage to deprivation data. The date of first recorded psychiatric presentation defined the ‘index date’. CYP may have a psychiatric condition recorded by a GP before their data is recorded in the CPRD-Aurum, for example at another general practice. To increase the likelihood that incident cases are captured, patients whose index date was less than 6 months from the date they registered at the practice were excluded. When missing, participants’ ethnicity was extracted from the HES dataset.

Follow up was defined as years since index date, and CYP with <1 year follow-up were excluded, resulting in 369,340 participants. These were followed from their index date until the earliest date of: death, transferring out of practice, end of data collection (5^th^ May 2021). The median follow-up was 6·3 years [IQR: 4·4 to 8·7]. For the present analysis, follow up was limited to 5 years.

### Psychiatric conditions

Psychiatric conditions were identified from CPRD-Aurum clinical codes recorded in primary care (a combination of SNOMED, Read and local EMIS codes). Code-lists mapped these to diagnostic and symptom concepts for WHO ICD-10 classifications of: a) autism spectrum and pervasive developmental disorders (F84); b) attention deficit and hyperkinetic disorders (F90); c) psychotic disorders (F20-31); d) depressive disorders (F32-39); e) anxiety disorders (F44-48); f) conduct and oppositional defiant disorder (F91); g) self-harm (X60-X84); h) eating disorders (F50-53); i) tic disorders and emotional, behavioural and social functioning disorders specific to childhood (F92 – 98); j) non-specific behavioural problems.^16^

A binary variable indicated whether each type of condition was recorded for a patient at their index presentation, or within the next 30 days. An individual could have multiple categories encoded (appendix pp3-4). Inclusion of symptom and diagnostic codes improves sensitivity for identifying mental illness; this approach aimed to include symptomatic presentations where a formal diagnosis may have been made at a later date in secondary care, and cases with diagnostic uncertainty.^17^ Previously published code lists were used where available (appendix p5). If previous code lists were unavailable or not recent, a clinician (MS), created new code lists by: 1) reviewing ICD10 to create a list of stub terms for symptoms and diagnoses, 2) using stubs to search text descriptors of CPRD codes 3) reviewing this list of codes for face vailidity.^18^ The final code lists were reviewed by a consultant psychiatrist in child and adolescent mental health (SG) to ensure all terms were correctly classified and had face validity. Codelists will be uploaded and freely available at https://clinicalcodes.rss.mhs.man.ac.uk/.

### Mental healthcare contact variables

At each year of follow up, three binary mental healthcare contact variables were coded from patients’ primary care records (appendix pp4,6): 1) GP contact related to psychiatric symptoms or diagnoses (included if they occurred >=30 days from the index presentation to avoid double counting the index presentation); 2) GP-issued prescription for psychotropic medications (antidepressants, antipsychotics, anxiety medications, mood stabilisers, ADHD medications); 3) Contact with specialist mental health services (psychiatrists, psychologists and child and adolescent mental health services) or referral for psychiatric symptoms or diagnoses.

### External clinical variables

Hospital admissions for self-harm were identified using linked HES inpatient register: any admission coded as ‘intentional self-harm’ (ICD-10 codes X60-84). Completed suicide was identified from ONS (including ‘undetermined’ intent: ICD-10 codes X60-84 and Y10-Y14).

### Covariates

Additional data were extracted on year of birth; gender (female, male, or indeterminate^19^); ethnicity according to ONS census categories (Black, South Asian, Mixed, Other and White); area-level deprivation (Index for Multiple Deprivation quintile -IMD); and geographical region (Appendix p3). These were selected because gender, socioeconomic status, and ethnicity are all associated with childhood trajectories of psychiatric symptoms,^6,11^ and may also influence service use by influencing help-seeking.^20,21^

### Statistical analysis

For our primary analysis the proportion with each type of healthcare contact was calculated yearly: overall, and by baseline covariates. Next, Group-Based Multi-Trajectory Models (GBMTM) identified groups of CYP with similar trajectories. GBMTM is an extension of group-based trajectory modelling to identify trajectory groupings for multiple-outcome processes (in this case, multiple types of healthcare contact).^22^ Separate models were fitted to allow for formation of 1-7 groups, each adjusted by year and year squared. A maximum of seven groups was allowed to retain interpretability, decrease vulnerability to identifying spurious groups and ensure analysis was computationally feasible. The optimal number of groups was identified based on: a) Akaike information criteria (AIC), b) Bayesian Information Criteria (BIC), c) entropy >0·7, d) group size >5% of total sample, e) odds of correct classification (OCC) >5 for all groups, and f) model interpretability. Following selection, if there were large standard errors and non-significant p-values for the quadratic or linear terms, a lower order model was tested.^23^

Once the optimal model was identified, CYP were classified into their most likely trajectory. Three analyses investigated the associations between trajectory group membership and baseline characteristics or subsequent clinical outcomes. First, CYPs’ most likely trajectory group membership was cross-tabulated with baseline characteristics to provide a descriptive overview of the groups, with chi-squared tests determining the statistical significance of any difference. Second, we considered whether potential healthcare inequalities were independent of other clinical characteristics at baseline. Therefore, we fitted multinomial logistic regression models with trajectory membership as the outcome variable, and demographic variables (ethnicity, gender, deprivation, and year of index presentation) and clinical characteristics expected to influence symptom course and clinical decision making (age at first presentation, comorbidity status and type of psychiatric condition at baseline) as the covariates..^8,10,11^

Finally, Cox regression models examined associations between trajectory group membership and time-to hospital admission for self-harm or suicide, referenced to the most common trajectory group. Censoring occurred at the earliest date of: event of interest, end of data collection, or death. We fitted unadjusted and adjusted survival models, with the latter assessing whether the association between trajectory membership and subsequent adverse event was independent of the risks associated with baseline covariates: psychiatric disorder at baseline, ethnicity, IMD quintile, age at baseline, and gender. The proportional hazard assumption was assessed by graphically inspecting the survival function.

### Sensitivity analysis

GBMTM accounts for partial follow-up using maximum likelihood estimation, assuming that attrition is not related to the outcome. To test the sensitivity of the model to this the GBMTM analysis was repeated for CYP with the full 5 years of follow-up but including inverse probability of missingness weights. These weights were estimated using a logistic regression model, with a missingness indicator as the dependent variable and baseline covariates as the independent variables. Additional, post-hoc analysis examined the influence of IMD quintile (most or least deprived) on associations between ethnicity and group membership (appendix p19).

Analyses used Stata v16 and GBMTM were fitted using the Traj package. Plots were created with the ggplot2 package in R.

## Results

369,340 CYP were included (49·0% girls; Table 1); median age at index presentation was 13·6 [IQR 8·4 - 16·7] years. The commonest baseline presentations were depressive symptoms (25·2%), anxiety symptoms (23·5%), or behavioural problems (20·9%). 285,267 CYP (77%) had ≥ 5 years follow-up (appendix p2); those with longer follow-up were slightly younger (appendix pp7-9).

### Likelihood of healthcare contacts following index presentation

**Figure 1:**
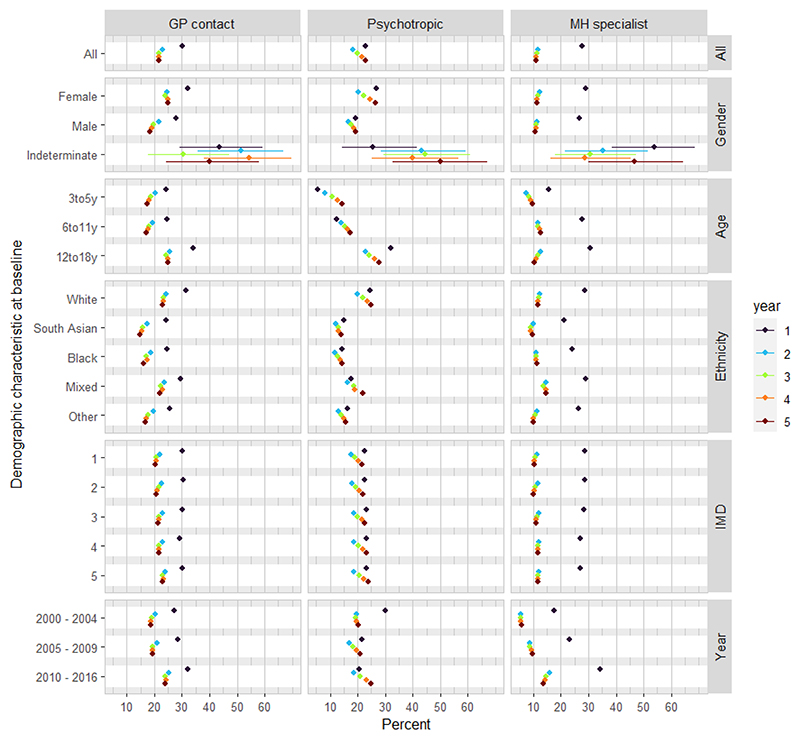
percentage with outcomes during each year following index presentation, by demographic characteristics Notes: Percent indicates % of sample with one or more: a) GP contacts for psychiatric symptoms/diagnoses, b) psychotropic prescriptions, c) mental health specialist contact within each complete year after the individual’s index presentation.

For each year after index presentation, most had none of the following: further GP contact for a psychiatric condition, psychotropic prescription, or mental health specialist contact (Figure 1). In the first year, only 30·1% had further GP contact and only 21·6% had GP contact in the fifth year (appendix pp11-12). In the first year, nearly a quarter (22·9%) received psychotropic prescriptions with stable rates over follow-up (e.g., 22·8% in fifth year; appendix pp13-14), whilst 27·8% had contact with specialist services which decreased to 11·0% in the fifth year (appendix pp15-16).

Girls were more likely than boys to have all types of contact over each year of follow-up with the greatest differences seen for GP contacts and psychotropic prescriptions (e.g. 32·3% girls vs 28·0% boys had subsequent GP consultations for psychiatric problems during year 1; Figure 1). Those whose gender was recorded as ‘indeterminate’ were most likely to see a mental health specialist. White ethnicity CYP had higher rates of GP contact and psychotropic prescription than South Asian, Black and other non-white ethnicities, and mixed ethnicity CYP had higher GP contact and psychotropic prescribing rates compared to Black, South Asian and other non-white ethnicities. Strikingly, service contact did not differ according to deprivation although some regional variation was seen (Figure 1, appendix p16).

Contact with specialists was more common for CYP presenting later in the study period: in Year 1, 34·2% of those presenting between 2010-2016 compared to 17·6% of those presenting 2000-2004.

**Figure 2:**
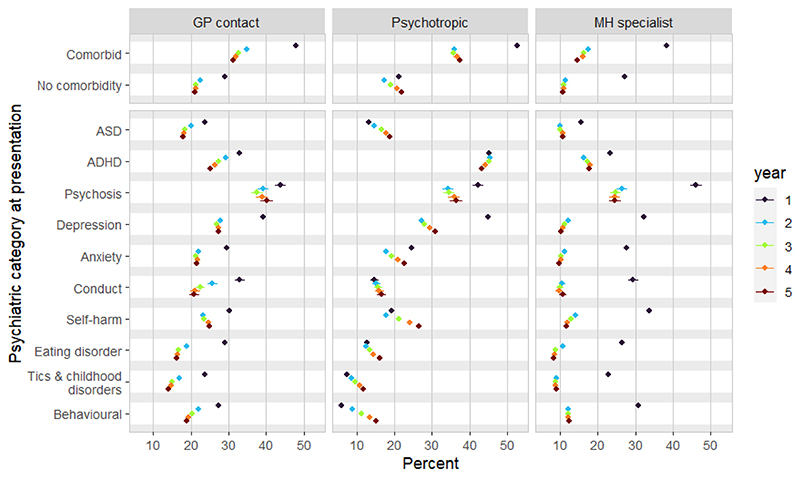
percentage with outcomes during each year following index presentation, by clinical characteristics Notes: Percent indicates % of sample with one or more: a) GP contacts for psychiatric symptoms/diagnoses, b) psychotropic prescriptions, c) mental health specialist contact within each complete year after the individual’s index presentation.

CYP with psychotic symptoms/diagnosis (1·2% of the sample) had the highest likelihood of further GP (43·8% year 1, 40·1% year 5; Figure 2) or specialist (46·1% year 1, 24·6% year 5) contact. Those with ADHD symptoms/diagnoses (7·1%) had the highest rates of psychotropic prescription (45·2% year 1, 43·3% year 5), followed by those with symptoms or diagnoses of depression (44·9% and 30·8%, respectively).

### Trajectory analysis

**Figure 3:**
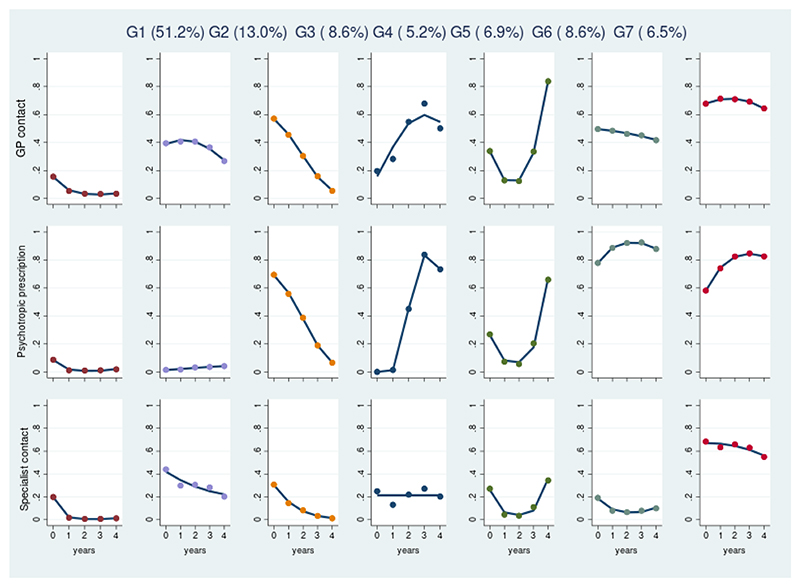
trajectory groups of GP contact, psychotropic prescriptions, and mental health specialist contact Notes: Y axis describes the model’s predicted service use trajectories in terms of the probability of group members a) having mental health-related contact with GP, b) receiving a prescription for a psychotropic, c) having contact with specialist mental health services during each year after index presentation. G1 = *low contact*, G2 = *moderate, non-pharmacological support*, G3 = *declining contact*, G4 = *year 4 escalating contact*, G5 = *year 5 escalating contact*, G6 = *prolonged GP contact*, G7 = *prolonged specialist contact*

The seven group GBMTM, with quadratic terms for year for all but two group/outcome combinations, had the best fit statistics. (least negative BIC and AIC), while retaining utility (group sizes >5%) and reasonable classification accuracy (entropy 0.75, average posterior probability >0.7) (Figure 3; full details of fit statistics in appendix pp17,18).

Group 1 (*low contact*, 51·2% of sample) had very low probabilities of further service use (GP contact for a psychiatric symptom/diagnosis, psychotropic prescription, and specialist service contact) throughout follow-up and was the largest group (Table 2). Boys were slightly over-represented in this group (54·4% compared to 51·0% of the whole sample, Table 2, appendix pp20-21), which also tended to be slightly younger (median 12·6 years, versus 13·6 in the whole sample). South Asian, Black, mixed ethnicity and other non-white ethnicity CYP were also over-represented (e.g. 3·9% Black, versus 3·2% of whole sample, Table 2). These associations remained after adjusting for clinical covariates and age (appendix p20-21). This group presented more commonly in earlier years, and in the least deprived neighbourhoods. CYP who initially presented with tics and other childhood-specific disorders such as separation anxiety were over-represented in this group (7·3% of group members vs 6·2% of whole sample), as were CYP presenting with symptoms/diagnosis of an eating disorder (4·6% of group members vs 4·1% of sample) and ASD (9·7% of group members, 8·7% of sample%). CYP presenting with depression, psychosis, self-harm, or ADHD were under-represented [appendix p23].

Group 2 (*moderate, non-pharmacological contact*, 13·0% of sample) had a moderate probability of ongoing GP or specialist mental health contacts, but low probability of psychotropic prescription. Of all the groups, this group had the greatest proportion of CYP with behavioural problems, ASD and conduct disorder symptoms. This group was also the youngest, had the highest proportion of boys, and presented in later years. (Table 2).

Group 3 (*declining contact*, 8·6%) had high rates of GP contact and prescriptions following initial presentation which dropped over 5 years. Compared with all groups, they were the oldest (median 16·9 years, IQR 14·7 – 18·1) and more likely to be girls (60·7%). CYP with depressive symptoms made up over half of this group (50·5%), compared to 27·3% of the whole cohort. Membership of this group was associated with white ethnicity and earlier years of presentation (Table 2, appendix p22).

Two smaller groups showed escalating mental healthcare use following initial presentation: group 4 (*year-4 escalating contact*, 5·2%, their GP contacts and prescriptions plateaued after year 4) and group 5 (*year-5 escalating contact*, 6·9%). Compared to the overall cohort, these were older at first presentation (median 14·9 years, group 4; 15·3, group 5). Girls and white CYP made up a larger percentage of this group than the sample as a whole. Of all groups, group 4 had the highest proportion of CYP who presented with self-harm (12·1%), while group 5 had a high proportion of CYP with anxiety symptoms (27·0% of group members, the highest of any group) and depressive symptoms (35·9% compared to 27.3% of the cohort).

Finally, two groups had high psychotropic drug prescription rates and high rates of GP contact (group 6 *prolonged GP contact*, 8·6%), with one group additionally having high specialist contacts (group 7 *prolonged specialist contact*, 6·5%). Of all groups, group 6 had the highest proportion of CYP with ADHD symptoms (21·7% of group members [appendix p23]), and group 7 had the highest proportion with psychotic symptoms (4·4% of group members, with 24·1% of CYP presenting with psychotic symptoms belonging to this group) [appendix p23]. Compared to the overall sample, both groups were older (median age 15·6 years for G6, 15·5 for G7, Table 2), and more likely to be of White ethnicity. CYP presenting in earlier years were over-represented in the *prolonged GP contact* group compared to the sample as a whole (25·8% presented 2000 – 2004 compared to 20·7% of cohort) while those presenting in later years were over-represented in the *prolonged specialist contact* group (70·1% of group members presented in 2010 - 2016, compared to 53·0% of cohort).

### Survival analysis

Beyond the five years after their index appointment (i.e. after their trajectory), compared with the ‘low-contact’ group, all groups had a higher rate of completed suicide or of self-harm requiring hospitalisation. Those in the *prolonged specialist contact* group had the highest rates, experiencing over 2·5 times the risk of suicide (HR = 2·67, 1·72-4·14) (n= 24; 0·006% of cohort); and over 2 times the risk of hospital admission for self-harm. These associations persisted after adjusting for covariates (Table 3).

### Sensitivity analyses

When trajectory modelling was repeated using only those with >= 5 years of follow-up, weighted to account for non-random attrition, similar trajectories were identified as in the main analysis, with cohort members assigned to groups in similar proportions (appendix p24).

Analysis code is available on request from the authors.

## Discussion

Just over half of children and young people attending primary care with psychological symptoms belonged to a *low contact* trajectory group with a had a low probability of further clinical contact (GP contact, prescriptions, or specialist care) each year after presentation: this was the most likely trajectory for all categories of psychiatric symptom. Potentially, this is reassuring inasmuch as it suggests symptoms may have resolved, initial contact was adequate, or further support has been accessed outside a medical setting. Alternatively, some CYP may still have unmet needs; have disengaged from services; or have not reengaged following unhelpful initial contact. Although many non-clinical factors can influence help-seeking for psychiatric symptoms by CYP and carers,^20,24^ five years of follow-up should allow us to see those with unresolved symptoms re-present in primary or secondary care.

Previous reports also suggest that most common mental health symptoms presenting in CYP do not persist long-term: in one English cohort, 68% of adolescents with depressive symptoms had symptoms confined to their teenage years and similarly, one in five CYP with psychopathology at aged 3 in an Irish population cohort had persistent symptoms in late childhood; persistence was more likely from early to late adolescence (41% of those with any psychopathology).^7,9^ While conditions such as psychosis, ASC and ADHD might be expected to cause more enduring difficulties, the help-seeking population we studied may not have met diagnostic thresholds, which could explain why the low contact trajectory was the most common group for CYP with symptoms in these categories.. Our ‘low contact’ group also had the lowest risk of subsequent hospitalisation for self-harm or completed suicide which supports the notion that they are *clinically* lower-risk.

Some CYP with ongoing clinical need are represented in the *Year 4-* and *Year 5-escalating contact* groups - 5·2% and 6·9% of the cohort, respectively. For these, interventions following initial GP contact may have been unhelpful, or they may have had more complex needs. Distinguishing CYP who benefit from early support to prevent deterioration versus those whose symptoms are likely to resolve without further help could usefully inform changes to service configurations and preventive practice, particularly within resource-limited contexts. The latter group may require reassurance and psychoeducation while the former may require more intensive intervention.^25,26^

In later years of the study period, membership of *low, declining*, and *prolonged GP contact* groups reduced, while membership increased for *prolonged specialist contact* group (with high psychotropic prescription throughout), and the percentage of CYP with specialist contacts was higher for those presenting in later years (almost two-times higher in the first year after presentation), consistent with recent trends in psychotropic prescriptions for CYP in primary care.^27^ CYP presenting to specialists in recent years may have a more complex, prolonged or severe clinical course requiring medications;^28^ or illness severity may have remained unchanged, but CYP receive more specialist long-term support and medication. Changes in service provision may result from an increasing emphasis on CYP mental health in the UK’s national health strategy, accompanied by increased funding for specialist services.^29^

Notably, CYP from Black, South Asian and other non-white ethnicities had lower rates of ongoing GP contact and psychotropic prescription than White CYP; there were similar, less pronounced differences for mixed ethnicity CYP. This adds to substantial evidence that people’s experiences and use of healthcare for psychiatric conditions in the UK varies according to ethnicity.^30^ Little is known about whether and how illness trajectories vary by ethnicity, making it difficult to determine whether illness trajectories influenced these findings. Asian and Black CYP who access CAMHS are more likely to be referred through education, social and other services rather than primary care.^21^ Our findings extend these findings and suggest that trajectories of mental healthcare use vary according to ethnicity for CYP who have accessed primary care services at presentation, raising questions about whether the initial primary care contact was experienced as helpful for non-white families and which other supports might be accessed, including educational or informal support.^31^ Rates of primary care contact are not lower for Black and South Asian CYP compared to White CYP so disparities appear specific to contact for mental health problems.^32^ Importantly, experiences of healthcare for psychiatric symptoms in childhood might influence service use pathways in adulthood when, for example, pathways to psychiatric care for psychosis involve higher rates of detention for Black and South Asian individuals compared to White patients.^33^

### Strengths and Limitations

Our trajectory approach was seen as valuable by our CYP advisory group and allowed us explicitly to describe heterogeneity in trajectories and use all outcome events within the follow-up period rather than measuring outcomes at discrete time points. We identified cases using both symptom and diagnostic codes reflecting the real-world primary care caseload which increases sensitivity for identifying cases of anxiety and depression.^17^ By examining shared service use trajectories of individuals with a broad range of mental health problems, we accounted for the interconnected nature of mental health symptoms in CYP, an approach that appears valuable given observed heterotypic associations between mental disorders in childhood and adulthood (across diagnostic categories).^12,14^ Future research could apply this analytical approach to specific conditions, for example examining prescribing trajectories for relevant medication classes.

However, there remain important limitations. Using a primary care database allowed us to examine real-world service use in a large, population-representative sample, capturing outcome events at any time during follow-up. However, we were not able to examine some outcomes important to CYP such as educational attainment, symptom severity, or social outcomes. Future research could examine a broader range of outcomes through linkage to educational and household datasets.^34^ Analyses over longer follow-up periods may also be valuable, for example examining whether early difficulties (e.g. psychotic symptoms or neurodevelopmental conditions) are a marker of vulnerability to later mental health difficulties. However, attrition from routine datasets might undermine the validity of trajectory modelling over longer time periods. We identified contacts with specialist services through GP records which may have led to under-ascertainment. However, secondary care datasets for mental health services in England are incomplete and specialist services routinely communicate assessment and treatment details to primary care; therefore, primary care data provided a more complete record. The data source may also overestimate the number of follow-up ‘GP contacts’ since some observations may have been administrative; or psychiatric symptoms may not have been the primary reason for consultation. There are also limitations to GBMTM: this identifies groups with similar outcome trajectories, but residual variation within trajectory groups potentially leads to misclassification of some individuals. In addition, GBMTM can identify spurious groups and, while we used a range of model adequacy criteria to mitigate this risk, repeating this analysis in different cohorts will be important to confirm the replicability of these groups.^35^ Finally, the approach to missing data relies on an assumption that data are missing at random, which may not be true for our dataset. Although we attempted to address this limitation using sensitivity analysis, non-random missingness may not have been fully captured in the variables available.

### Conclusions

We find that most CYP presenting to primary care with mental, behavioural, or neurodevelopmental symptoms do not receive prolonged medical care and that, over time young people show very mixed mental health service use. The majority ‘low contact’ group also show the lowest rate of hospitalisations for self-harm or suicide, which may offer some reassurance to families and CYP. We measured clinical need indirectly via clinical contacts; future research, using linked datasets to examine a broader range of educational, social, and clinical outcomes, may help determine whether CYP with *low contact* trajectories have unmet needs. Given that CYP are primarily brought to services by parents/caregivers, future research should examine how home environment influences which CYP access mental healthcare and when, and the course of service use. Other sources of support, including schools, community-based and informal networks, are important elements in initial and continued service access for young people and more research is needed to understand their role in mental health prevention and treatment for CYP.

## Supplementary Material

Supplementary material

## Figures and Tables

**Table 1:** cohort characteristics

Baseline characteristic	N	%
**Total**	369,340	
***Age at presentation (y)*** Median [IQR]	13.6 [8.4 - 16.7]	
***Gender***		
Female	180,863	49.0
Male	188,438	51.0
Indeterminate	39	0.0
Missing	0	
***Ethnicity***		
White	290,125	78.6
South Asian	9,161	2.5
Black	10,418	2.8
Mixed	8,115	2.2
Other	8,587	2.3
Missing	42,934	11.6
***Mental health category at index presentation***		
ASD	32,094	8.7
ADHD	26,279	7.1
Psychosis	4,276	1.2
Depression	93,114	25.2
Anxiety	86,596	23.5
Conduct disorder	4,343	1.2
Self-harm (>6y only)	30,312	8.2
Eating disorder	14,286	3.9
Tics and childhood-specific disorders	21,551	5.8
Behavioural	77,070	20.9
Missing	0	
Comorbidity (Multiple categories at baseline)	19,413	5.3
***IMD quintile***		
1 (least deprived)	63,209	17.1
2	66,078	17.9
3	66,779	18.1
4	76,729	20.8
5 (most deprived)	96,107	26.0
Missing	438	0.1
***Region***		
North East	14,418	3.9
North West	72,543	19.6
Yorkshire And The Humber	13,502	3.7
East Midlands	8,820	2.4
West Midlands	59,907	16.2
East of England	16,936	4.6
South West	46,998	12.7
South Central	48,764	13.2
London	52,721	14.3
South East Coast	34,301	9.3
Missing	430	0.1
***Year of index presentation***		
2000 - 2004	76,592	20.7
2005 - 2009	97,101	26.3
2010 - 2016	195,647	53.0

Notes: percentages are column percentages

**Table 2:** Baseline characteristics of the 7 latent class trajectory groups

	*Trajectory group*							
	*All* *sample*	1: low contact n= 207,985	2: moderate non- pharmacological support N= 43,836	3: declining contact, N=25,469	4: Year 4 escalating contact, N = 18,277	5: Year 5 escalating contact, N = 18,139	6: Prolonged GP contact, N= 32,147	7: Prolonged specialist contact, N= 23,487
***Age***								
*Med (IQR)*	*13.6 (8.4 - 16.7)*	12.6 (7.7 – 16.3)	10.8 (7.0 – 14.3)	16.9 (14.7 – 18.1)	14.9 (10.4 – 16.7)	15.3 (11.9 – 17.4)	15.6 (9.5 – 17.8)	15.5 12.3 – 17.4)
***Gender (%)***								
*Female*	*49.0*	45.6	43.2	60.7	59.0	60.8	51.7	56.5
*Male*	*51.0*	54.4	56.8	39.3	41.0	39.2	48.3	43.5
***Ethnicity (%)***								
*White*	*88.9*	86.8	88.4	92.6	92.5	92.1	93.5	90.7
*South Asian*	*2.8*	3.5	2.5	2.0	1.5	1.9	1.4	2.0
*Black*	*3.2*	3.9	3.4	1.9	2.0	1.9	1.7	2.4
*Mixed*	*2.5*	2.6	3.1	1.8	2.3	2.2	1.9	2.6
*Other*	*2.6*	3.2	2.6	1.8	1.7	1.9	1.5	2.2
***IMD quintile (%)***								
*1 (least* *deprived)*	*17.1*	17.6	16.5	17.0	16.2	16.5	16.5	16.6
*2*	*17.9*	18.2	17.7	18.1	17.4	17.2	17.2	17.2
*3*	*18.1*	18.1	17.8	18.4	18.3	18.0	18.1	18.3
*4*	*20.8*	20.7	20.5	20.4	20.8	21.1	21.1	18.1
*5 (most deprived)*	*26.0*	25.3	27.3	25.9	27.2	27.2	27.0	26.0
***Year of presentation (%)***								
*2000 to 2004*	*20.7*	22.5	12.4	27.7	16.1	19.5	25.8	10.5
*2005 to 2009*	*26.3*	28.2	22.6	25.5	24.2	25.7	25.9	19.4
*2010 to 2016*	*53.0*	49.3	64.9	46.7	59.7	54.8	48.3	70.1
***Mental health category at index appointment (%)***								
*ASD*	*8.7*	9.7	**10.7**	3.5	7.3	5.5	8.8	5.4
*ADHD*	*7.1*	4.6	5.6	8.7	6.8	4.4	**21.7**	13.2
*psychosis*	*1.3*	0.8	1.1	1.3	1.0	1.1	1.3	**4.4**
*depression*	*27.3*	20.4	13.9	**50.5**	28.4	35.9	35.5	36.4
*anxiety*	*24.8*	23.9	19.2	26.1	24.7	**27.0**	20.9	23.9
*conduct*	*1.3*	1.2	**1.6**	0.8	0.9	0.9	1.0	0.9
*self-harm*	*9.1*	7.9	7.3	9.4	**12.1**	10.5	5.5	10.5
*eating disorder*	*4.1*	**4.6**	3.3	2.8	2.9	3.0	1.9	3.5
*Tics etc*	*6.2*	**7.3**	6.8	2.2	3.5	3.7	2.8	2.5
*Behavioural*	*22.3*	23.1	**34.8**	6.0	17.6	14.9	10.7	12.1
*Comorbid*	*5.3*	3.3	4.0	10.8	5.0	6.3	9.6	**11.9**

Data reflect column-wise percentages (excluding those missing baseline variable), apart from for age. Frequency and cross-tabulation with region are shown in supplementary table 6. Bold is used to highlight which group is most over-represented for each category.

**Table 3:** Results from Cox regression analysis, examining the association between trajectory group and hospital admission for intentional self-harm, or death by suicide

Outcome	Trajectory group	Number of events	Rate*	HR [95% CI]	aHR [95% CI]**
Hospital admission for intentional self-harm, ICD-10 X60-X84	*1: low contact*	4,620	3.28 [3.19, 3.37]	Ref	Ref
	*2: moderate non-pharmacological support*	1,257	5.57 [5.27, 5.89]	1.65 [1.55, 1.76]	1.50 [1.41, 1.60]
	*3: declining contact*	658	3.59 [3.33, 3.88]	1.11 [1.02, 1.20]	1.03 [0.95, 1.13]
	*4: Year 4 escalating contact*	612	5.91 [5.46, 6.40]	1.77 [1.63, 1.93]	1.54 [1.42, 1.68]
	*5: Year 5 escalating contact*	715	6.34 [5.89, 6.82]	1.92 [1.77, 2.07]	1.68 [1.55, 1.83]
	*6: Prolonged GP contact*	1,052	4.72 [4.45, 5.02]	1.45 [1.36, 1.55]	1.37 [1.28, 1.47]
	*7: Prolonged specialist contact*	815	7.46 [6.96, 7.99]	2.19 [2.03, 2.36]	1.98 [1.83, 2.14]
					
Suicide	*1: low contact*	121	0.08 [0.07, 0.10]	Ref	Ref
	*2: moderate non-pharmacological support*	29	0.13 [0.09, 0.18]	1.55 [1.03, 2.33]	1.97 [1.27, 3.06]
	*3: declining contact*	22	0.12 [0.08, 0.18]	1.38 [0.88, 2.17]	1.25 [0.77, 2.03]
	*4: Year 4 escalating contact*	16	0.15 [0.09, 0.25]	1.83 [1.08, 3.08]	1.90 [1.08, 3.33]
	*5: Year 5 escalating contact*	20	0.17 [0.11, 0.27]	2.05 [1.28, 3.30]	2.02 [1.22, 3.36]
	*6: Prolonged GP contact*	26	0.11 [0.08, 0.17]	1.34 [0.88, 2.04]	1.33 [0.84, 2.13]
	*7: Prolonged specialist contact*	24	0.21 [0.14, 0.32]	2.67 [1.72, 4.14]	2.24 [1.38, 3.63]

* Rate = per 1,000 person years

** **Adjusted for: presenting symptom/disorder at baseline, ethnicity, IMD quintile, age at baseline, gender**

## Data Availability

The clinical codes, data management, and analysis code used in this study are available on request from the corresponding author. Access to raw data can be requested via application to the Clinical Practice Research Datalink. The criteria for applying for these data are available on the Clinical Practice Research Datalink website: https://cprd.com/.

## Research in context

### Evidence before this study

We searched PubMed from inception to April 3, 2023, for articles published in English using search terms related to (a) mental health and neurodevelopmental conditions (e.g. “mental health” OR “mental illness” OR depress* OR anxiety) AND (b) (trajectory OR prognos* OR outcome) AND (c) (child* OR youth OR adolesc*). Several studies have examined trajectories of psychopathological symptoms in population cohorts of children and adolescents in the UK, and Ireland. This evidence suggests that heterogeneity in symptom trajectories is the norm; different symptom clusters have distinct and developmentally sensitive trajectories; common difficulties frequently co-occur; and symptom trajectories limited to childhood or adolescence are common. Other previous research has studied outcomes for CYP with psychiatric conditions by examining average clinical and educational measures at discrete time points following baseline assessment. These sources of evidence suggest that on average CYP with psychiatric symptoms have poorer clinical and educational outcomes than healthy peers, but with substantial heterogeneity that may be masked by analytical approaches that rely on averaged outcomes at discrete follow-up points. We did not identify any studies examining outcomes for clinical populations of CYP presenting to primary care, or examining healthcare contacts as an outcome.

### Added value of this study

Using a cohort of CYP (aged 3 – 18 years) identified through an English primary care database, we explored 5-year trajectories of healthcare use in a real-world clinical population who initially presented with symptoms of psychiatric conditions. We identified seven distinct trajectories, describing heterogenous service use patterns in this population. The largest group (51·2% of the sample) had low rates of ongoing mental healthcare use and this group had the lowest risk of hospitalisation for self-harm and death by suicide. CYP were more likely to be members of this group if they were younger, male and from non-white ethnicities.

### Implications of all the available evidence

A substantial body of evidence suggests that long-term educational, clinical, and social outcomes are worse for CYP with psychiatric conditions than for healthy peers. This has concerning public health implications in light of recent increases in the number of CYP presenting to healthcare services with psychiatric symptoms. However, we found that the majority of CYP presenting to primary care with psychiatric symptoms do not have persistent mental health service contacts. One explanation for this could be that many help-seeking CYP have transient symptom trajectories that differ from previously studied cohorts.
