# Supplementary material for: Five-year mental health outcomes for children and young people presenting to GPs in England with psychiatric symptoms
Source: Lancet Psychiatry. Author manuscript; Available in PMC 2026 Jan 30. (PMC7618697; doi:10.1016/S2215-0366(24)00038-5)
Supplement: Supplementary material [file EMS212021-supplement-Supplementary_material.docx]

**Contents**

Page 2 **Supplementary Figure 1: inclusion flowchart**

Page 3 **eMethods 1: additional details on variable ascertainment**

Page 5 **eMethods 3: source of clinical codelists**

Page 6 **eMethods 2: process for identifying ADHD medications**

Page 7 **Supplementary Table 1: characteristics of sample by length of follow-up**

Page 10 **Supplementary table 2: number and percentage with GP contacts for each year of follow-up, by baseline characteristics**

Page 12 **Supplementary table 3: number and percentage with psychotropic prescription for each year of follow-up, by baseline characteristics**

Page 14 **Supplementary table 4: number and percentage with specialist service contact for each year of follow-up, by baseline characteristics**

Page 16 **Supplementary Figure 2: percentage outcomes by GP practice region**

Page 17 **Supplementary Table 5: fit statistics for group-based trajectory models with different numbers of groups**

Page 19 **Supplementary table 6: cross-tabulation of region with group membership**

Page 20 **Supplementary table 7: results of multinomial logistic regression analysis. Average probability of group membership by gender, ethnicity, IMD quintile and year of presentation**

Page 22 **Supplementary table 8: average probability of group membership by ethnicity for least and most deprived IMD quintile**

Page 23  **Supplementary table 9: probability of group membership by baseline clinical characteristics**

Page 24 **Supplementary figure 3: sensitivity analysis – group-based trajectory modelling**

Page 25 **GRoLTS checklist**

Page 26 **References**


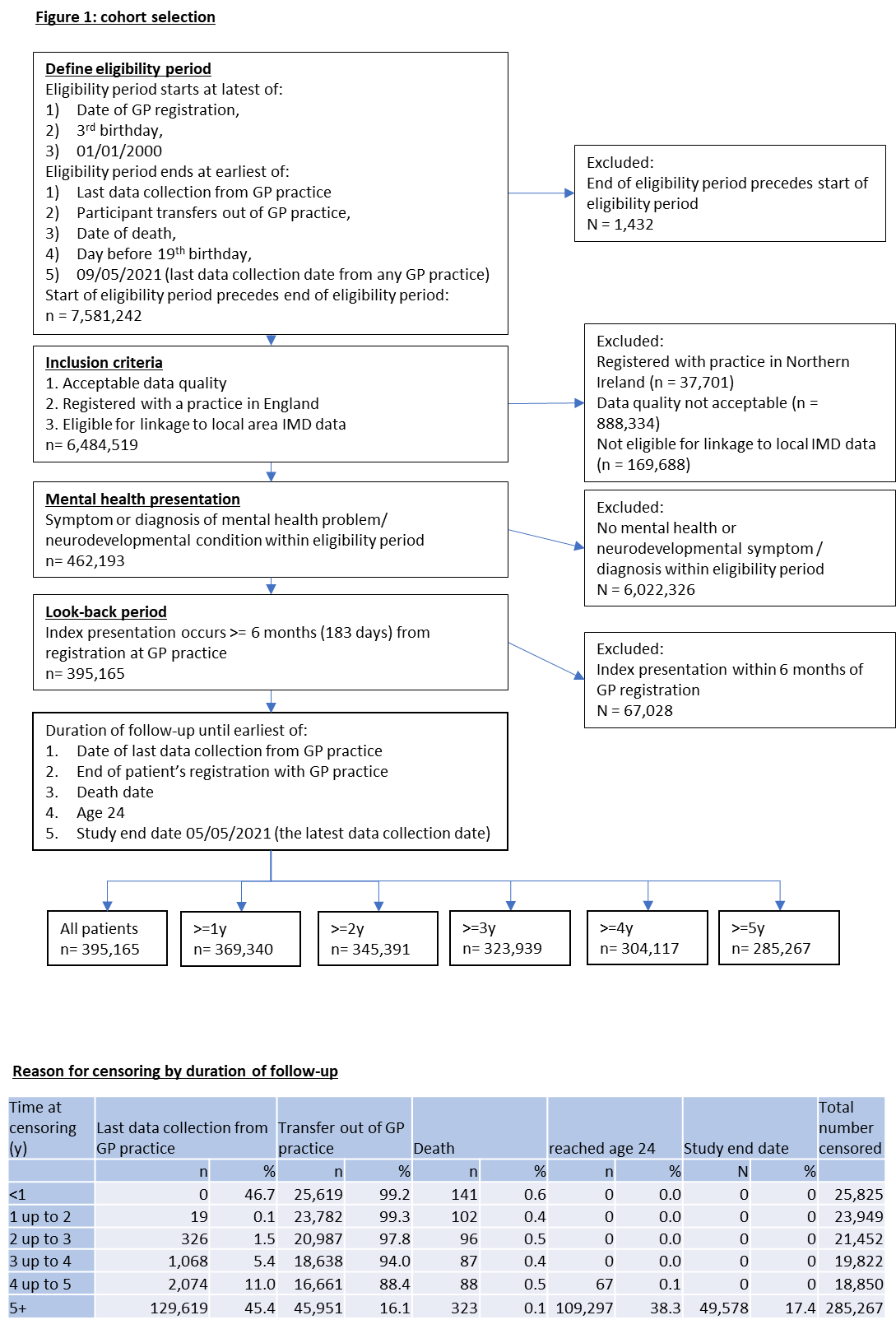


Note: percentages are given out of total number censored within each year of follow-up

**eMethods 1: additional information on variables used in cohort selection and analysis**

| Variable | Description |
| --- | --- |
| Acceptable data quality | CPRD provides a patient-level measure of data quality or ‘acceptability’ that is based on recording and internal consistency of key variables including date of birth, practice registration date and transfer out of practice date. |
| ***Baseline characteristics*** | |
| Age at presentation | Derived from date of birth recorded in CPRD data.  Exact dates of birth are not included in CPRD in order to maintain anonymity. For patients in CPRD aged >= 16 year of birth is recorded and for those aged <16 years, month and year of birth are recorded. If month of birth was missing, we assigned June as the month of birth. We assigned day of birth as the 15th day of the month if month of birth was presenting in the dataset, and 30th day if month of birth was missing.  For analyses, age at first presentation was categorised into: 3–5 years (up to the day before 6th birthday), 6-11 years, and 12-18 years corresponding to preschool, middle childhood and adolescent stages of development. |
| Ethnicity | Ethnicity was derived from CPRD data where it is recorded using clinical codes. For each patient, codes were used to categorise ethnicity as: White (including White British, Irish and other European ethnicities), Black, South Asian, Mixed (indicating that ethnicity was recorded as mixed, rather than that multiple categories were recorded), and other (including Chinese and Arab). Ethnicity recording in UK primary care data is frequently incomplete, although recording has improved since the introduction of financial incentives in 2006.^1^ To supplement information from CPRD we extracted ethnicity as recorded in HES APC, Outpatient or emergency care datasets, whichever had the most recent ethnicity code, since ethnicity recording is more complete in these datasets. Where multiple different ethnicity codes were recorded, we used the most frequently recorded, and if multiple categories were coded with equal frequency we used the most recent code.  Ethnicity was used as a factor variable in analysis. |
| IMD quintile | Index of Multiple Deprivation (IMD) quintile for participants’ postcodes was used as a measure of relative deprivation with quintile 1 being the least deprived, and 5 the most. The Indices of Multiple Deprivation (IMD) are based on indicators of deprivation across multiple domains (including income, employment, health, education) that are weighted and combined to provide an indication of the relative deprivation of different neighbourhoods in England.^2^ |
| Gender | Gender was derived from CPRD data and relates to self-reported gender or gender inferred during clinical encounters which does not exactly map to either biological sex or self-identified gender.^3^ Participants were classified into female, male and indeterminate genders. |
| Region | GP practices were categorised into 10 geographical regions. |
| Mental health category | The type of mental health problem at baseline was derived from CPRD Aurum medcodes – observations that are recorded by primary care practitioners during consultations. These codes are based on a combination of several different structured coding systems: SNOMED CT (UK Edition), Read and local EMIS Web codes. CPRD-Aurum data is provided with a data dictionary mapping these codes to relevant descriptions and we used code-lists to map these codes to diagnoses and symptoms related to different categories of mental health problem (WHO ICD-10 categorisation. These were:   1. autism spectrum and pervasive developmental disorders (ICD-10 F84); 2. attention deficit and hyperkinetic disorders (F90); 3. psychotic disorders (F20-31); 4. depressive disorders (F32-39 including dysthymia); 5. anxiety and stress-related disorders (F44-48); 6. conduct disorder and oppositional defiant disorder (F91); 7. tic disorders and emotional, behavioural and social functioning disorders specific to childhood (F92 – 98 including selective mutism, separation anxiety); 8. self-harm (X60-X84) indicated by self-injury with suicidal or self-injurious intent, or by poisoning/overdose regardless of recorded intent. Only self-harm codes for individuals aged >=6 years were included since intentional self-harm in younger age groups is rare and may be difficult to distinguish from accidental self-injury or self-poisoning. 9. eating disorders (F50-53); 10. non-specific symptoms or diagnoses. Codes indicating personality disorders were included in this category since the validity of personality disorder diagnoses in this age group is questionable.   For analysis, a binary variable was created for each category of mental health problem. Participants were classified as having a mental health problem from a specific category if a relevant symptom or diagnostic code was present within 30 days of the date of cohort entry. Children and young people could therefore be classified as having multiple categories of mental health problem. Including both symptom and diagnostic codes as evidence of a mental health problem has several advantages: children and young people presenting in primary care with mental health problems may not meet diagnostic criteria for a mental disorder but those with subthreshold symptoms may have specific needs and may be at increased risk of future mental illness; the recording of diagnostic and symptom codes may depend on factors other than clinical presentation (for example financial incentives based on clinical coding) making it difficult to differentiate between subthreshold disorders and those meeting diagnostic criteria; and including symptom codes can improve sensitivity for identifying cases of mental illness compared to using only diagnostic codes.^4,5^ |
| Comorbidity | A binary variable for comorbidity at baseline was included. CYP with codes for multiple categories of mental health problem within 30 days of first presentation were designated as having a comorbidity. |
| **Outcomes** | |
| GP contacts | We identified mental health-related contacts with the GP occurring during the follow-up period and for analysis these were incorporated into a binary variable indicating the presence/absence of further contacts within each year following index presentation. Observation codes indicating a symptom, diagnosis or service use for a mental health problem were assumed to indicate contact for a mental health-related problem. We excluded codes indicating a historical diagnosis or symptom and those occurring in the first 30 days after index presentation to avoid double counting the index presentation. |
| Psychotropic prescriptions | Receipt of a prescription for a psychotropic medication was identified from GP-issued prescriptions recorded in CPRD. Previously published code lists were used to identify prescriptions for antidepressants, antipsychotics, anxiety medications (including benzodiazepines) and mood stabilisers. For medications used in the treatment of ADHD no code list was available for CPRD Aurum so MS created a new code list according to the process described in appendix p6 and this was reviewed by SG. A binary variable was created indicating whether a child or young person received one or more prescriptions for a psychotropic medication within each year of follow-up. |
| Contact with mental health specialist services | Contact with specialist mental health services was identified from CPRD observation and referral codes (observation codes included, for example “seen by psychiatrist” or “referral to child and adolescent psychiatry service”). A binary variable indicated whether each child or young person had had contact with specialist services within each year of follow-up. |

**eMethods 3: sources of clinical codelists**

| MH category | Codelist sources |
| --- | --- |
| asd (and pervasive developmental disorders) | Cybulski 2021^6^, Hagberg 2017^7^ + supplemented with new codelist |
| adhd (and hyperkinetic) | Cybulski 2021^6^, Houghton 2018^8^ + supplemented with new codelist |
| anxiety & depression | Abel 2019 ^9^ |
| Psychosis | Abel 2019 ^9^ |
| depressive disorders | Abel 2019 ^9^ |
| eating disorder | Abel 2019 ^9^ |
| neurotic - anxiety disorders | Abel 2019 ^9^ |
| Tics and childhood-specific emotional and behavioural disorders | new codelist generated |
| conduct disorder and ODD | new codelist generated |
| self-harm/suicide/suicidal ideation | Cybulski 2021^6^ |

**eMethods 4: process for identifying ADHD medication codes**

Prescription information is coded using the dictionary of medications and devices, within SNOMED.

1. Include codes with BNF chapter code 0404000: CNS stimulants and drugs used for ADHD (largely incomplete in EMIS dictionary): 61 codes
2. Develop glossary of terms based on BNF chapter 0404000, NICE guidelines for management of ADHD in children, previous codelists, and Maudsley prescribing guidelines.* ^10,11^
3. Search EMIS terms/drug substance names/product names for generic drug names: + 95 codes
4. Search EMIS terms/drug substance names/product names for brand drug names: + 4 additional codes
5. Exclude non-oral preparations (clonidine) (9 codes removed)
6. Exclude caffeine, pitolisant (stimulants not recommended or licenced for ADHD) (4 codes removed)
7. Manual review of codes by clinician, removal of bogus codes (1 removed)
8. Compare to previously published codelist, add any additional substances,^12^ no changes.

* Search terms (brackets indicate term contains another search term, and is therefore obsolete):

amfetamine amphetamine atomoxetine clonidine (dexamfetamine) dexmethylphenidate (dexamphetamine) (dextroamphetamine) guanfacine (lisdexamfetamine) methylphenidate modafinil

adderall amfexa atomaid concerta durophet delmosart dexedrine elvanse equasym focalin intuniv matoride medikinet metyrol provigil ritalin strattera tranquilyn vyvanse xaggitin xenidate

**Table 1: characteristics of sample by length of follow-up**

| Baseline characteristic |  |  | >=1 year f/u | | >=2y f/u | | >=3y fu | | >=4y fu | | >=5y | |
| --- | --- | --- | --- | --- | --- | --- | --- | --- | --- | --- | --- | --- |
|  | N | % | N | % | N | % | N | % | N | % | N | % |
| Total | 395,165 |  | 369,340 |  | 345,391 |  | 323,939 |  | 304,117 |  | 285,267 |  |
| *Age at presentation*  Median [IQR] | 13.7 [8.4 – 16.8] |  | 13.6 [8.4 - 16.7] |  | 13.5 [8.4 - 16.6] |  | 13.4 [8.3 - 16.6] |  | 13.3 [8.2 - 16.5] |  | 13.1 [8.2 - 16.5] |  |
| *Gender* |  |  |  |  |  |  |  |  |  |  |  |  |
| Female | 194,846 | 49.3 | 180,863 | 49.0 | 167,728 | 48.6 | 155,815 | 48.1 | 144,542 | 47.5 | 133,937 | 47.0 |
| Male | 200,280 | 50.7 | 188,438 | 51.0 | 177,626 | 51.4 | 168,088 | 51.9 | 159,540 | 52.5 | 151,300 | 53.0 |
| Indeterminate | 39 | 0.0 | 39 | 0.0 | 37 | 0.0 | 36 | 0.0 | 35 | 0.0 | 30 | 0.0 |
| Missing | 0 |  | 0 |  | 0 |  |  |  | 0 |  |  |  |
| *Ethnicity* |  |  |  |  |  |  |  |  |  |  |  |  |
| White | 309,961 | 78.4 | 290,125 | 78.6 | 271,729 | 78.7 | 255,228 | 78.8 | 239,817 | 78.9 | 224,983 | 78.9 |
| South Asian | 9,700 | 2.5 | 9,161 | 2.5 | 8,683 | 2.5 | 8,230 | 2.5 | 7,820 | 2.6 | 7,446 | 2.6 |
| Black | 11,348 | 2.9 | 10,418 | 2.8 | 9,501 | 2.8 | 8,724 | 2.7 | 8,048 | 2.7 | 7,465 | 2.6 |
| Mixed | 8,782 | 2.2 | 8,115 | 2.2 | 7,509 | 2.2 | 6,978 | 2.2 | 6,478 | 2.1 | 6,032 | 2.1 |
| Other | 9,372 | 2.4 | 8,587 | 2.3 | 7,921 | 2.3 | 7,313 | 2.3 | 6,813 | 2.2 | 6,333 | 2.2 |
| Missing | 46,002 | 11.6 | 42,934 | 11.6 | 40,048 | 11.6 | 37,466 | 11.6 | 35,141 | 11.6 | 33,008 | 11.6 |
| *Mental health category at baseline* |  |  |  |  |  |  |  |  |  |  |  |  |
| ASD | 33,843 | 8.6 | 32,094 | 8.7 | 30,369 | 8.8 | 28,960 | 8.9 | 27,595 | 9.1 | 26,257 | 9.2 |
| ADHD | 28,036 | 7.1 | 26,279 | 7.1 | 24,686 | 7.2 | 23,294 | 7.2 | 22,144 | 7.3 | 20,985 | 7.4 |
| psychosis | 4,651 | 1.2 | 4,276 | 1.2 | 3,941 | 1.1 | 3,636 | 1.1 | 3,375 | 1.1 | 3,143 | 1.1 |
| depression | 100,826 | 25.5 | 93,114 | 25.2 | 85,768 | 24.8 | 79,224 | 24.5 | 73,217 | 24.1 | 67,540 | 23.7 |
| anxiety | 91,627 | 23.2 | 86,596 | 23.5 | 81,718 | 23.7 | 76,915 | 23.7 | 72,230 | 23.8 | 67,796 | 23.8 |
| conduct disorder | 4,622 | 1.2 | 4,343 | 1.2 | 4,077 | 1.2 | 3,866 | 1.2 | 3,682 | 1.2 | 3,476 | 1.2 |
| self-harm (>6y only) | 33,569 | 8.5 | 30,312 | 8.2 | 27,865 | 8.1 | 25,563 | 7.9 | 23,542 | 7.7 | 21,548 | 7.6 |
| eating disorder | 15,211 | 3.9 | 14,286 | 3.9 | 13,351 | 3.9 | 12,506 | 3.9 | 11,703 | 3.9 | 10,998 | 3.9 |
| tics and childhood-specific disorders | 22,774 | 5.8 | 21,551 | 5.8 | 20,438 | 5.9 | 19,456 | 6.0 | 18,593 | 6.1 | 17,753 | 6.2 |
| Behavioural | 82,295 | 20.8 | 77,070 | 20.9 | 72,245 | 20.9 | 68,246 | 21.1 | 64,521 | 21.2 | 61,042 | 21.4 |
| Missing | 0 |  | 0 |  | 0 |  | 0 |  | 0 |  | 0 |  |
| Comorbid | 21,007 | 5.3 | 19,413 | 5.3 | 18,000 | 5.2 | 16,721 | 5.2 | 15,546 | 5.1 | 14,411 | 5.1 |
| *IMD quintile* |  |  |  |  |  |  |  |  |  |  |  |  |
| 1 (least deprived) | 67,211 | 17.0 | 63,209 | 17.1 | 59,364 | 17.2 | 55,832 | 17.2 | 52,516 | 17.3 | 49,347 | 17.3 |
| 2 | 70,321 | 17.8 | 66,078 | 17.9 | 62,051 | 18.0 | 58,295 | 18.0 | 54,721 | 18.0 | 51,303 | 18.0 |
| 3 | 71,368 | 18.1 | 66,779 | 18.1 | 62,341 | 18.1 | 58,452 | 18.0 | 54,833 | 18.0 | 51,438 | 18.0 |
| 4 | 82,290 | 20.8 | 76,729 | 20.8 | 71,716 | 20.8 | 67,190 | 20.7 | 63,035 | 20.7 | 59,103 | 20.7 |
| 5 (most deprived) | 103,482 | 26.2 | 96,107 | 26.0 | 89,531 | 25.9 | 83,819 | 25.9 | 78,696 | 25.9 | 73,792 | 25.9 |
| Missing | 493 | 0.1 | 438 | 0.1 | 388 | 0.1 | 351 | 0.1 | 316 | 0.1 | 284 | 0.1 |
| *Region* |  |  |  |  |  |  |  |  |  |  |  |  |
| North East | 15,289 | 3.9 | 14,418 | 3.9 | 13,643 | 4.0 | 12,908 | 4.0 | 12,161 | 4.0 | 11,467 | 4.0 |
| North West | 77,290 | 19.6 | 72,543 | 19.6 | 68,402 | 19.8 | 64,687 | 20.0 | 61,448 | 20.2 | 58,223 | 20.4 |
| Yorkshire And The Humber | 14,470 | 3.7 | 13,502 | 3.7 | 12,653 | 3.7 | 11,803 | 3.6 | 10,926 | 3.6 | 10,084 | 3.5 |
| East Midlands | 9,509 | 2.4 | 8,820 | 2.4 | 8,196 | 2.4 | 7,547 | 2.3 | 6,883 | 2.3 | 6,293 | 2.2 |
| West Midlands | 64,043 | 16.2 | 59,907 | 16.2 | 56,160 | 16.3 | 52,861 | 16.3 | 49,779 | 16.4 | 46,775 | 16.4 |
| East of England | 18,014 | 4.6 | 16,936 | 4.6 | 15,897 | 4.6 | 14,924 | 4.6 | 13,880 | 4.6 | 12,871 | 4.5 |
| South West | 50,184 | 12.7 | 46,998 | 12.7 | 44,026 | 12.8 | 41,177 | 12.7 | 38,549 | 12.7 | 36,156 | 12.7 |
| South Central | 52,064 | 13.2 | 48,764 | 13.2 | 45,477 | 13.2 | 42,693 | 13.2 | 40,137 | 13.2 | 37,725 | 13.2 |
| London | 56,938 | 14.4 | 52,721 | 14.3 | 48,607 | 14.1 | 45,199 | 14.0 | 42,135 | 13.9 | 39,282 | 13.8 |
| South East Coast | 36,892 | 9.3 | 34,301 | 9.3 | 31,942 | 9.3 | 29,781 | 9.2 | 27,902 | 9.2 | 26,107 | 9.2 |
| Missing | 472 | 0.1 | 430 | 0.1 | 388 | 0.1 | 359 | 0.1 | 317 | 0.1 | 284 | 0.1 |
| *Year of presentation* |  |  |  |  |  |  |  |  |  |  |  |  |
| 2000 to 2004 | 81,643 | 20.7 | 76,592 | 20.7 | 71,811 | 20.8 | 67,493 | 20.8 | 63,789 | 21.0 | 60,343 | 21.2 |
| 2005 to 2009 | 104,381 | 26.4 | 97,101 | 26.3 | 90,321 | 26.2 | 84,582 | 26.1 | 79,568 | 26.2 | 75,055 | 26.3 |
| 2010 to 2016 | 209,141 | 52.9 | 195,647 | 53.0 | 183,259 | 53.1 | 171,864 | 53.1 | 160,760 | 52.9 | 149,869 | 52.5 |
| missing | 0 |  | 0 |  | 0 |  | 0 |  | 0 |  | 0 |  |

**Supplementary table 2: number and percentage with GP contacts for each year of follow-up, by baseline characteristics**

| Baseline characteristic | Year 1 | | | | Year 2 | | | | Year 3 | | | | Year 4 | | | | Year 5 | | | |
| --- | --- | --- | --- | --- | --- | --- | --- | --- | --- | --- | --- | --- | --- | --- | --- | --- | --- | --- | --- | --- |
|  | N | % [95% CI] | | | N | % [95% CI] | | | N | % [95% CI] | | | N | % [95% CI] | | | N | % [95% CI] | | |
| All | 111173 | 30.1 | [30.0 | - 30.3] | 79,585 | 23.0 | [22.9 | - 23.2] | 70,715 | 21.8 | [21.7 | - 22.0] | 66,321 | 21.8 | [21.7 | - 22.0] | 61,462 | 21.6 | [21.4 | - 21.7] |
| Female | 58383 | 32.3 | [32.1 | - 32.5] | 41326 | 24.6 | [24.4 | - 24.9] | 37501 | 24.1 | [23.9 | - 24.3] | 35991 | 24.9 | [24.7 | - 25.1] | 33482 | 25.0 | [24.8 | - 25.2] |
| Male | 52773 | 28.0 | [27.8 | - 28.2] | 38240 | 21.5 | [21.3 | - 21.7] | 33203 | 19.8 | [19.6 | - 19.9] | 30311 | 19.0 | [18.8 | - 19.2] | 27968 | 18.5 | [18.3 | - 18.7] |
| Indeterminate | 17 | 43.6 | [29.1 | - 59.3] | 19 | 51.4 | [35.7 | - 66.8] | 11 | 30.6 | [17.8 | - 47.2] | 19 | 54.3 | [37.9 | - 69.8] | 12 | 40.0 | [24.3 | - 58.1] |
| 3to5y | 11905 | 24.2 | [23.8 | - 24.6] | 9419 | 20.3 | [20.0 | - 20.7] | 8217 | 18.8 | [18.4 | - 19.1] | 7511 | 18.1 | [17.7 | - 18.5] | 6891 | 17.4 | [17.1 | - 17.8] |
| 6to11y | 25647 | 24.7 | [24.4 | - 25.0] | 19105 | 19.4 | [19.2 | - 19.6] | 17004 | 18.1 | [17.8 | - 18.3] | 15921 | 17.7 | [17.5 | - 18.0] | 14775 | 17.2 | [17.0 | - 17.5] |
| 12to18y | 25647 | 34.1 | [33.9 | - 34.3] | 51061 | 25.5 | [25.3 | - 25.7] | 45494 | 24.4 | [24.3 | - 24.6] | 42889 | 24.9 | [24.6 | - 25.1] | 39796 | 24.9 | [24.7 | - 25.1] |
| White | 90892 | 31.3 | [31.2 | - 31.5] | 66198 | 24.4 | [24.2 | - 24.5] | 59343 | 23.3 | [23.1 | - 23.4] | 55701 | 23.2 | [23.1 | - 23.4] | 52003 | 23.1 | [22.9 | - 23.3] |
| South Asian | 2222 | 24.3 | [23.4 | - 25.1] | 1500 | 17.3 | [16.5 | - 18.1] | 1289 | 15.7 | [14.9 | - 16.5] | 1210 | 15.5 | [14.7 | - 16.3] | 1114 | 15.0 | [14.2 | - 15.8] |
| Black | 2562 | 24.6 | [23.8 | - 25.4] | 1785 | 18.8 | [18.0 | - 19.6] | 1495 | 17.1 | [16.4 | - 17.9] | 1406 | 17.5 | [16.7 | - 18.3] | 1204 | 16.1 | [15.3 | - 17.0] |
| Mixed | 2384 | 29.4 | [28.4 | - 30.4] | 1779 | 23.7 | [22.7 | - 24.7] | 1554 | 22.3 | [21.3 | - 23.3] | 1495 | 23.1 | [22.1 | - 24.1] | 1332 | 22.1 | [21.1 | - 23.2] |
| Other | 2192 | 25.5 | [24.6 | - 26.5] | 1552 | 19.6 | [18.7 | - 20.5] | 1291 | 17.7 | [16.8 | - 18.5] | 1159 | 17.0 | [16.1 | - 17.9] | 1065 | 16.8 | [15.9 | - 17.8] |
| 1 | 19166 | 30.3 | [30.0 | - 30.7] | 13116 | 22.1 | [21.8 | - 22.4] | 11553 | 20.7 | [20.4 | - 21.0] | 10911 | 20.8 | [20.4 | - 21.1] | 10037 | 20.3 | [20.0 | - 20.7] |
| 2 | 20181 | 30.5 | [30.2 | - 30.9] | 14111 | 22.7 | [22.4 | - 23.1] | 12589 | 21.6 | [21.3 | - 21.9] | 11518 | 21.1 | [20.7 | - 21.4] | 10675 | 20.8 | [20.5 | - 21.2] |
| 3 | 20193 | 30.2 | [29.9 | - 30.6] | 14324 | 23.0 | [22.7 | - 23.3] | 12670 | 21.7 | [21.3 | - 22.0] | 11903 | 21.7 | [21.4 | - 22.1] | 11053 | 21.5 | [21.1 | - 21.9] |
| 4 | 22501 | 29.3 | [29.0 | - 29.7] | 16491 | 23.0 | [22.7 | - 23.3] | 14606 | 21.7 | [21.4 | - 22.1] | 13660 | 21.7 | [21.4 | - 22.0] | 12755 | 21.6 | [21.3 | - 21.9] |
| 5 | 28980 | 30.2 | [29.9 | - 30.4] | 21440 | 24.0 | [23.7 | - 24.2] | 19208 | 22.9 | [22.6 | - 23.2] | 18251 | 23.2 | [22.9 | - 23.5] | 16881 | 22.9 | [22.6 | - 23.2] |
| 2000 - 2004 | 20893 | 27.3 | [27.0 | - 27.6] | 14635 | 20.4 | [20.1 | - 20.7] | 12981 | 19.2 | [18.9 | - 19.5] | 11956 | 18.7 | [18.4 | - 19.1] | 11299 | 18.7 | [18.4 | - 19.0] |
| 2005 - 2009 | 27579 | 28.4 | [28.1 | - 28.7] | 18911 | 20.9 | [20.7 | - 21.2] | 16429 | 19.4 | [19.2 | - 19.7] | 15393 | 19.4 | [19.1 | - 19.6] | 14462 | 19.3 | [19.0 | - 19.6] |
| 2010 - 2016 | 62701 | 32.1 | [31.8 | - 32.3] | 46039 | 25.1 | [24.9 | - 25.3] | 41305 | 24.0 | [23.8 | - 24.2] | 38972 | 24.2 | [24.0 | - 24.5] | 35701 | 23.8 | [23.6 | - 24.0] |
| ASD | 7609 | 23.7 | [23.3 | - 24.2] | 6117 | 20.1 | [19.7 | - 20.6] | 5343 | 18.5 | [18.0 | - 18.9] | 5031 | 18.2 | [17.8 | - 18.7] | 4683 | 17.8 | [17.4 | - 18.3] |
| ADHD | 8642 | 32.9 | [32.3 | - 33.5] | 7250 | 29.4 | [28.8 | - 29.9] | 6363 | 27.3 | [26.8 | - 27.9] | 5838 | 26.4 | [25.8 | - 27.0] | 5281 | 25.2 | [24.6 | - 25.8] |
| Psychosis | 1874 | 43.8 | [42.3 | - 45.3] | 1544 | 39.2 | [37.7 | - 40.7] | 1365 | 37.5 | [36.0 | - 39.1] | 1311 | 38.8 | [37.2 | - 40.5] | 1260 | 40.1 | [38.4 | - 41.8] |
| Depression | 36470 | 39.2 | [38.9 | - 39.5] | 23947 | 27.9 | [27.6 | - 28.2] | 21329 | 26.9 | [26.6 | - 27.2] | 19953 | 27.3 | [26.9 | - 27.6] | 18437 | 27.3 | [27.0 | - 27.6] |
| Anxiety | 25557 | 29.5 | [29.2 | - 29.8] | 17920 | 21.9 | [21.7 | - 22.2] | 16395 | 21.3 | [21.0 | - 21.6] | 15757 | 21.8 | [21.5 | - 22.1] | 14685 | 21.7 | [21.4 | - 22.0] |
| Conduct | 1433 | 33.0 | [31.6 | - 34.4] | 1047 | 25.7 | [24.4 | - 27.0] | 869 | 22.5 | [21.2 | - 23.8] | 777 | 21.1 | [19.8 | - 22.5] | 726 | 20.9 | [19.6 | - 22.3] |
| Self-harm | 9162 | 30.2 | [29.7 | - 30.8] | 6494 | 23.3 | [22.8 | - 23.8] | 5989 | 23.4 | [22.9 | - 24.0] | 5785 | 24.6 | [24.0 | - 25.1] | 5357 | 24.9 | [24.3 | - 25.4] |
| Eating disorder | 4158 | 29.1 | [28.4 | - 29.9] | 2516 | 18.9 | [18.2 | - 19.5] | 2079 | 16.6 | [16.0 | - 17.3] | 1929 | 16.5 | [15.8 | - 17.2] | 1797 | 16.3 | [15.7 | - 17.0] |
| Tics & childhood disorders | 5118 | 23.8 | [23.2 | - 24.3] | 3477 | 17.0 | [16.5 | - 17.5] | 2922 | 15.0 | [14.5 | - 15.5] | 2743 | 14.8 | [14.3 | - 15.3] | 2497 | 14.1 | [13.6 | - 14.6] |
| Behavioural | 21086 | 27.4 | [27.1 | - 27.7] | 15980 | 22.1 | [21.8 | - 22.4] | 13861 | 20.3 | [20.0 | - 20.6] | 12470 | 19.3 | [19.0 | - 19.6] | 11497 | 18.8 | [18.5 | - 19.2] |
| No comorbidity | 101,867 | 29.1 | [29.0 | - 29.3] | 73296 | 22.4 | [22.3 | - 22.5] | 65254 | 21.2 | [21.1 | - 21.4] | 61366 | 21.3 | [21.1 | - 21.4] | 56973 | 21.0 | [20.9 | - 21.2] |
| Comorbid | 9,306 | 47.9 | [47.2 | - 48.6] | 6289 | 34.9 | [34.3 | - 35.6] | 5461 | 32.7 | [32.0 | - 33.4] | 4955 | 31.9 | [31.2 | - 32.6] | 4489 | 31.2 | [30.4 | - 31.9] |

**Supplementary table 3: number and percentage with psychotropic prescription for each year of follow-up, by baseline characteristics**

| Baseline characteristic | Year 1 | | | | Year 2 | | | | Year 3 | | | | Year 4 | | | | Year 5 | | | |
| --- | --- | --- | --- | --- | --- | --- | --- | --- | --- | --- | --- | --- | --- | --- | --- | --- | --- | --- | --- | --- |
|  | N | % [95% CI] | | | N | % [95% CI] | | | N | % [95% CI] | | | N | % [95% CI] | | | N | % [95% CI] | | |
| All | 51294 | 22.9 | [22.8 | - 23.1] | 70166 | 18.4 | [18.3 | - 18.5] | 62498 | 19.9 | [19.8 | - 20.1] | 58810 | 21.5 | [21.3 | - 21.6] | 54571 | 22.8 | [22.7 | - 23.0] |
| Female | 48415 | 26.8 | [26.6 | - 27.0] | 33968 | 20.3 | [20.1 | - 20.4] | 34779 | 22.3 | [22.1 | - 22.5] | 35623 | 24.7 | [24.4 | - 24.9] | 35680 | 26.6 | [26.4 | - 26.9] |
| Male | 36262 | 19.2 | [19.1 | - 19.4] | 29590 | 16.7 | [16.5 | - 16.8] | 29734 | 17.7 | [17.5 | - 17.9] | 29711 | 18.6 | [18.4 | - 18.8] | 29396 | 19.4 | [19.2 | - 19.6] |
| Indeterminate | 10 | 25.6 | [14.4 | - 41.4] | 16 | 43.2 | [28.5 | - 59.4] | 16 | 44.4 | [29.3 | - 60.7] | 14 | 40.0 | [25.3 | - 56.7] | 15 | 50.0 | [32.8 | - 67.2] |
| 3to5y | 2769 | 5.6 | [5.4 | - 5.8] | 3740 | 8.1 | [7.8 | - 8.3] | 4687 | 10.7 | [10.4 | - 11.0] | 5310 | 12.8 | [12.5 | - 13.1] | 5733 | 14.5 | [14.2 | - 14.9] |
| 6to11y | 12845 | 12.4 | [12.2 | - 12.6] | 13791 | 14.0 | [13.8 | - 14.2] | 14501 | 15.4 | [15.2 | - 15.7] | 14815 | 16.5 | [16.2 | - 16.7] | 14900 | 17.3 | [17.1 | - 17.6] |
| 12to18y | 69073 | 32.0 | [31.8 | - 32.2] | 46043 | 23.0 | [22.8 | - 23.1] | 45341 | 24.4 | [24.2 | - 24.6] | 45223 | 26.2 | [26.0 | - 26.4] | 44458 | 27.8 | [27.6 | - 28.0] |
| White | 71317 | 24.6 | [24.4 | - 24.7] | 54451 | 20.0 | [19.9 | - 20.2] | 55552 | 21.8 | [21.6 | - 21.9] | 56415 | 23.5 | [23.4 | - 23.7] | 56192 | 25.0 | [24.8 | - 25.2] |
| South Asian | 1392 | 15.2 | [14.5 | - 15.9] | 1059 | 12.2 | [11.5 | - 12.9] | 1064 | 12.9 | [12.2 | - 13.7] | 1030 | 13.2 | [12.4 | - 13.9] | 1056 | 14.2 | [13.4 | - 15.0] |
| Black | 1508 | 14.5 | [13.8 | - 15.2] | 1117 | 11.8 | [11.1 | - 12.4] | 1109 | 12.7 | [12.0 | - 13.4] | 1115 | 13.9 | [13.1 | - 14.6] | 1086 | 14.6 | [13.8 | - 15.4] |
| Mixed | 1441 | 17.8 | [16.9 | - 18.6] | 1235 | 16.5 | [15.6 | - 17.3] | 1301 | 18.6 | [17.8 | - 19.6] | 1236 | 19.1 | [18.1 | - 20.1] | 1319 | 21.9 | [20.8 | - 22.9] |
| Other | 1408 | 16.4 | [15.6 | - 17.2] | 1039 | 13.1 | [12.4 | - 13.9] | 1026 | 14.0 | [13.3 | - 14.8] | 1029 | 15.1 | [14.3 | - 16.0] | 991 | 15.7 | [14.8 | - 16.6] |
| 1 | 14286 | 22.6 | [22.3 | - 22.9] | 10573 | 17.8 | [17.5 | - 18.1] | 10669 | 19.1 | [18.8 | - 19.4] | 10729 | 20.4 | [20.1 | - 20.8] | 10693 | 21.7 | [21.3 | - 22.0] |
| 2 | 14878 | 22.6 | [22.3 | - 22.8] | 11123 | 17.9 | [17.6 | - 18.2] | 11254 | 19.3 | [19.0 | - 19.6] | 11229 | 20.5 | [20.2 | - 20.9] | 11226 | 21.9 | [21.5 | - 22.2] |
| 3 | 15409 | 23.1 | [22.8 | - 23.4] | 11561 | 18.5 | [18.2 | - 18.9] | 11725 | 20.1 | [19.7 | - 20.4] | 11886 | 21.7 | [21.3 | - 22.0] | 11666 | 22.7 | [22.3 | - 23.0] |
| 4 | 17704 | 23.1 | [22.8 | - 23.4] | 13413 | 18.7 | [18.4 | - 19.0] | 13547 | 20.2 | [19.9 | - 20.5] | 13832 | 21.9 | [21.6 | - 22.3] | 13727 | 23.2 | [22.9 | - 23.6] |
| 5 | 22284 | 23.2 | [22.9 | - 23.5] | 16831 | 18.8 | [18.5 | - 19.1] | 17258 | 20.6 | [20.3 | - 20.9] | 17602 | 22.4 | [22.1 | - 22.7] | 17718 | 24.0 | [23.7 | - 24.3] |
| 2000 - 2004 | 23033 | 30.1 | [29.8 | - 30.4] | 14038 | 19.6 | [19.3 | - 19.8] | 13113 | 19.4 | [19.1 | - 19.7] | 12612 | 19.8 | [19.5 | - 20.1] | 12236 | 20.3 | [20.0 | - 20.6] |
| 2005 - 2009 | 21068 | 21.7 | [21.4 | - 22.0] | 15285 | 16.9 | [16.7 | - 17.2] | 15423 | 18.2 | [18.0 | - 18.5] | 15626 | 19.6 | [19.4 | - 19.9] | 15687 | 20.9 | [20.6 | - 21.2] |
| 2010 - 2016 | 40586 | 20.7 | [20.6 | - 20.9] | 34251 | 18.7 | [18.5 | - 18.9] | 35993 | 20.9 | [20.8 | - 21.1] | 37110 | 23.1 | [22.9 | - 23.3] | 37168 | 24.8 | [24.6 | - 25.0] |
| ASD | 4234 | 13.2 | [12.8 | - 13.6] | 4464 | 14.7 | [14.3 | - 15.1] | 4797 | 16.6 | [16.1 | - 17.0] | 4955 | 18.0 | [17.5 | - 18.4] | 4962 | 18.9 | [18.4 | - 19.4] |
| ADHD | 11875 | 45.2 | [44.6 | - 45.8] | 11217 | 45.4 | [44.8 | - 46.1] | 10542 | 45.3 | [44.6 | - 45.9] | 9780 | 44.2 | [43.5 | - 44.8] | 9078 | 43.3 | [42.6 | - 43.9] |
| Psychosis | 1808 | 42.3 | [40.8 | - 43.8] | 1353 | 34.3 | [32.9 | - 35.8] | 1254 | 34.5 | [33.0 | - 36.1] | 1211 | 35.9 | [34.3 | - 37.5] | 1148 | 36.5 | [34.9 | - 38.2] |
| Depression | 41797 | 44.9 | [44.6 | - 45.2] | 23414 | 27.3 | [27.0 | - 27.6] | 22110 | 27.9 | [27.6 | - 28.2] | 21572 | 29.5 | [29.1 | - 29.8] | 20821 | 30.8 | [30.5 | - 31.2] |
| Anxiety | 21243 | 24.5 | [24.3 | - 24.8] | 14591 | 17.9 | [17.6 | - 18.1] | 14891 | 19.4 | [19.1 | - 19.6] | 15170 | 21.0 | [20.7 | - 21.3] | 15360 | 22.7 | [22.3 | - 23.0] |
| Conduct | 640 | 14.7 | [13.7 | - 15.8] | 621 | 15.2 | [14.2 | - 16.4] | 607 | 15.7 | [14.6 | - 16.9] | 590 | 16.0 | [14.9 | - 17.2] | 575 | 16.5 | [15.3 | - 17.8] |
| Self-harm | 5830 | 19.2 | [18.8 | - 19.7] | 4966 | 17.8 | [17.4 | - 18.3] | 5409 | 21.2 | [20.7 | - 21.7] | 5697 | 24.2 | [23.7 | - 24.8] | 5726 | 26.6 | [26.0 | - 27.2] |
| Eating disorder | 1831 | 12.8 | [12.3 | - 13.4] | 1676 | 12.6 | [12.0 | - 13.1] | 1695 | 13.6 | [13.0 | - 14.2] | 1699 | 14.5 | [13.9 | - 15.2] | 1789 | 16.3 | [15.6 | - 17.0] |
| Tics & childhood disorders | 1583 | 7.4 | [7.0 | - 7.7] | 1752 | 8.6 | [8.2 | - 9.0] | 1872 | 9.6 | [9.2 | - 10.0] | 2035 | 10.9 | [10.5 | - 11.4] | 2111 | 11.9 | [11.4 | - 12.4] |
| Behavioural | 4711 | 6.1 | [6.0 | - 6.3] | 6407 | 8.9 | [8.7 | - 9.1] | 7709 | 11.3 | [11.1 | - 11.5] | 8670 | 13.4 | [13.2 | - 13.7] | 9232 | 15.1 | [14.8 | - 15.4] |
| No comorbidity | 74487 | 21.3 | [21.2 | - 21.4] | 57123 | 17.5 | [17.3 | - 17.6] | 58548 | 19.1 | [18.9 | - 19.2] | 59662 | 20.7 | [20.5 | - 20.8] | 59705 | 22.0 | [21.9 | - 22.2] |
| Comorbid | 10200 | 52.5 | [51.8 | - 53.2] | 6451 | 35.8 | [35.1 | - 36.5] | 5981 | 35.8 | [35.1 | - 36.5] | 5686 | 36.6 | [35.8 | - 37.3] | 5386 | 37.4 | [36.6 | - 38.2] |

**Supplementary table 4: number and percentage with specialist service contact for each year of follow-up, by baseline characteristics**

| Baseline characteristic | Year 1 | | | | Year 2 | | | | Year 3 | | | | Year 4 | | | | Year 5 | | | |
| --- | --- | --- | --- | --- | --- | --- | --- | --- | --- | --- | --- | --- | --- | --- | --- | --- | --- | --- | --- | --- |
|  | N | % [95% CI] | | | N | % [95% CI] | | | N | % [95% CI] | | | N | % [95% CI] | | | N | % [95% CI] | | |
| All | 31 | 27.8 | [27.7 | - 28.0] | 29 | 11.8 | [11.7 | - 11.9] | 27 | 11.2 | [11.1 | - 11.4] | 24 | 11.2 | [11.1 | - 11.3] | 29 | 11.0 | [10.9 | - 11.2] |
| Female | 52524 | 29.0 | [28.8 | - 29.3] | 20573 | 12.3 | [12.1 | 12.4] | 18009 | 11.6 | [11.4 | - 11.7] | 16426 | 11.4 | [11.2 | - 11.5] | 15063 | 11.3 | [11.1 | - 11.4] |
| Male | 50288 | 26.7 | [26.5 | - 26.9] | 20180 | 11.4 | [11.2 | - 11.5] | 18382 | 10.9 | [10.8 | - 11.1] | 17565 | 11.0 | [10.9 | - 11.2] | 16413 | 10.9 | [10.7 | - 11.0] |
| Indeterminate | 21 | 53.9 | [38.3 | - 68.7] | 13 | 35.1 | [21.6 | - 51.6] | 11 | 30.6 | [17.8 | - 47.2] | 10 | 28.6 | [16.1 | - 45.4] | 14 | 46.7 | [29.9 | - 64.2] |
| 3 to5y | 7751 | 15.7 | [15.4 | - 16.1] | 3490 | 7.5 | [7.3 | - 7.8] | 3690 | 8.4 | [8.2 | - 8.7] | 3830 | 9.2 | [8.9 | - 9.5] | 3874 | 9.8 | [9.5 | - 10.1] |
| 6 to11y | 28901 | 27.8 | [27.6 | - 28.1] | 11596 | 11.8 | [11.6 | - 12.0] | 11129 | 11.8 | [11.6 | - 12.1] | 11224 | 12.5 | [12.3 | - 12.7] | 10851 | 12.6 | [12.4 | - 12.9] |
| 12 to18y | 66181 | 30.6 | [30.4 | - 30.8] | 25680 | 12.8 | [12.7 | - 13.0] | 21583 | 11.6 | [11.5 | - 11.7] | 18947 | 11.0 | [10.8 | - 11.1] | 16765 | 10.5 | [10.3 | - 10.6] |
| White | 82820 | 28.6 | [28.4 | - 28.7] | 33660 | 12.4 | [12.3 | - 12.5] | 30308 | 11.9 | [11.8 | - 12.0] | 28392 | 11.8 | [11.7 | - 12.0] | 26290 | 11.7 | [11.6 | - 11.8] |
| South Asian | 1938 | 21.2 | [20.3 | - 22.0] | 860 | 9.9 | [9.3 | - 10.6] | 739 | 9.0 | [8.4 | - 9.6] | 712 | 9.1 | [8.5 | - 9.8] | 731 | 9.8 | [9.2 | - 10.5] |
| Black | 2501 | 24.0 | [23.2 | - 24.8] | 1061 | 11.2 | [10.6 | - 11.8] | 970 | 11.1 | [10.5 | - 11.8] | 890 | 11.1 | [10.4 | - 11.8] | 837 | 11.2 | [10.5 | - 12.0] |
| Mixed | 2342 | 28.9 | [27.9 | - 29.9] | 1088 | 14.5 | [13.7 | - 15.3] | 944 | 13.5 | [12.8 | - 14.4] | 947 | 14.6 | [13.8 | - 15.5] | 883 | 14.6 | [13.8 | - 15.6] |
| Other | 2264 | 26.4 | [25.4 | - 27.3] | 903 | 11.4 | [10.7 | - 12.1] | 792 | 10.8 | [10.1 | - 11.6] | 687 | 10.1 | [9.4 | - 10.8] | 637 | 10.1 | [9.3 | - 10.8] |
| 1 | 18151 | 28.7 | [28.4 | - 29.1] | 6802 | 11.5 | [11.2 | - 11.7] | 6026 | 10.8 | [10.5 | - 11.1] | 5521 | 10.5 | [10.3 | - 10.8] | 5084 | 10.3 | [10.0 | - 10.6] |
| 2 | 18850 | 28.5 | [28.2 | - 28.9] | 7186 | 11.6 | [11.3 | - 11.8] | 6316 | 10.8 | [10.6 | - 11.1] | 5707 | 10.4 | [10.2 | - 10.7] | 5179 | 10.1 | [9.8 | - 10.4] |
| 3 | 18848 | 28.2 | [27.9 | - 28.6] | 7436 | 11.9 | [11.7 | - 12.2] | 6546 | 11.2 | [11.0 | - 11.5] | 6116 | 11.2 | [10.9 | - 11.4] | 5665 | 11.0 | [10.8 | - 11.3] |
| 4 | 20829 | 27.2 | [26.8 | - 27.5] | 8610 | 12.0 | [11.8 | - 12.3] | 7744 | 11.5 | [11.3 | - 11.8] | 7352 | 11.7 | [11.4 | - 11.9] | 6865 | 11.6 | [11.4 | - 11.9] |
| 5 | 26039 | 27.1 | [26.8 | - 27.4] | 10679 | 11.9 | [11.7 | - 12.1] | 9717 | 11.6 | [11.4 | - 11.8] | 9261 | 11.8 | [11.5 | - 12.0] | 8665 | 11.7 | [11.5 | - 12.0] |
| 2000 - 2004 | 12612 | 17.6 | [17.3 | - 17.9] | 12236 | 5.4 | [5.2 | - 5.5] | 13475 | 5.5 | [5.3 | - 5.6] | 3847 | 5.5 | [5.3 | - 5.7] | 3690 | 5.9 | [5.7 | - 6.1] |
| 2005 - 2009 | 15626 | 23.2 | [23.0 | - 23.5] | 15687 | 8.6 | [8.5 | - 8.8] | 22539 | 8.7 | [8.5 | - 8.9] | 7801 | 9.4 | [9.2 | - 9.6] | 7326 | 9.8 | [9.6 | - 10.0] |
| 2010 - 2016 | 37110 | 34.2 | [33.9 | - 34.4] | 37168 | 15.9 | [15.7 | - 16.1] | 66819 | 14.8 | [14.6 | - 14.9] | 29118 | 14.3 | [14.2 | - 14.5] | 25386 | 13.7 | [13.6 | - 13.9] |
| ASD | 4991 | 15.6 | [15.2 | - 16.0] | 3030 | 10.0 | [9.7 | - 10.3] | 2927 | 10.1 | [9.8 | - 10.5] | 2983 | 10.8 | [10.5 | - 11.2] | 2841 | 10.8 | [10.5 | - 11.2] |
| ADHD | 6156 | 23.4 | [22.9 | - 23.9] | 4020 | 16.3 | [15.8 | - 16.8] | 4006 | 17.2 | [16.7 | - 17.7] | 3974 | 18.0 | [17.5 | - 18.5] | 3746 | 17.9 | [17.3 | - 18.4] |
| Psychosis | 1973 | 46.1 | [44.7 | - 47.6] | 1041 | 26.4 | [25.1 | - 27.8] | 900 | 24.8 | [23.4 | - 26.2] | 825 | 24.4 | [23.0 | - 25.9] | 774 | 24.6 | [23.2 | - 26.2] |
| Depression | 29931 | 32.1 | [31.9 | - 32.5] | 10495 | 12.2 | [12.0 | - 12.5] | 8827 | 11.1 | [10.9 | - 11.4] | 7849 | 10.7 | [10.5 | - 11.0] | 6868 | 10.2 | [9.9 | - 10.4] |
| Anxiety | 23926 | 27.6 | [27.3 | - 27.9] | 9135 | 11.2 | [11.0 | - 11.4] | 7859 | 10.2 | [10.0 | - 10.4] | 7360 | 10.2 | [10.0 | - 10.4] | 6566 | 9.7 | [9.5 | - 9.9] |
| Conduct | 1277 | 29.4 | [28.1 | - 30.8] | 433 | 10.6 | [9.7 | - 11.6] | 384 | 9.9 | [9.0 | - 10.9] | 362 | 9.8 | [8.9 | - 10.8] | 371 | 10.7 | [9.7 | - 11.7] |
| Self-harm | 10223 | 33.7 | [33.2 | - 34.3] | 3961 | 14.2 | [13.8 | - 14.6] | 3298 | 12.9 | [12.5 | - 13.3] | 2809 | 11.9 | [11.5 | - 12.4] | 2549 | 11.8 | [11.4 | - 12.3] |
| Eating disorder | 3769 | 26.4 | [25.7 | - 27.1] | 1421 | 10.6 | [10.1 | - 11.2] | 1111 | 8.9 | [8.4 | - 9.4] | 1017 | 8.7 | [8.2 | - 9.2] | 922 | 8.4 | [7.9 | - 8.9] |
| Tics & childhood disorders | 4943 | 22.9 | [22.4 | - 23.5] | 1872 | 9.2 | [8.8 | - 9.6] | 1728 | 8.9 | [8.5 | - 9.3] | 1631 | 8.8 | [8.4 | - 9.2] | 1605 | 9.0 | [8.6 | - 9.5] |
| Behavioural | 23669 | 30.7 | [30.4 | - 31.0] | 8761 | 12.1 | [11.9 | - 12.4] | 8283 | 12.1 | [11.9 | - 12.4] | 7899 | 12.2 | [12.0 | - 12.5] | 7527 | 12.3 | [12.1 | - 12.6] |
| No comorbidity | 95399 | 27.3 | [27.1 | - 27.4] | 37606 | 11.5 | [11.4 | - 11.6] | 33681 | 11.0 | [10.9 | - 11.1] | 31489 | 10.9 | [10.8 | - 11.0] | 29372 | 10.8 | [10.7 | - 11.0] |
| Comorbid | 7434 | 38.3 | [37.6 | - 39.0] | 3160 | 17.6 | [17.0 | - 18.1] | 2721 | 16.3 | [15.7 | - 16.8] | 2512 | 16.2 | [15.6 | - 16.8] | 2118 | 14.7 | [14.1 | - 15.3] |

**Figure 2: percentage outcomes by GP practice region**


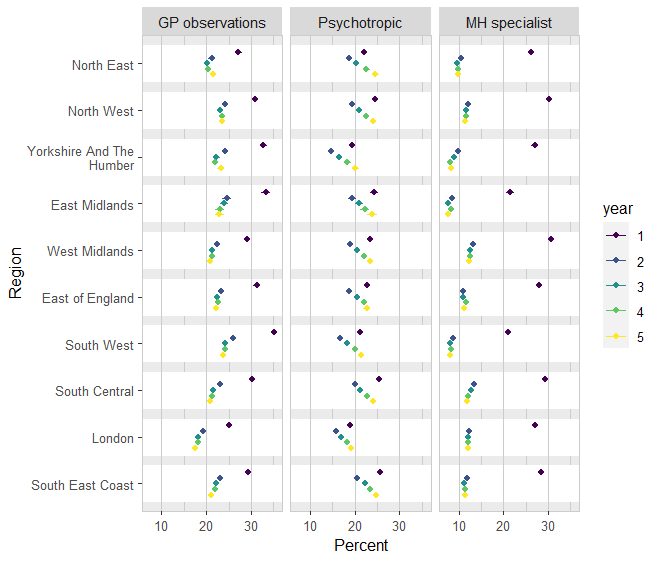


**Table 5: fit statistics for group-based trajectory models with different numbers of groups**

| N of groups | Parameter order | % membership per group | BIC 1 (N=4884162) | BIC 2 (N=369340) | AIC | Entropy | OCC per group | AvePP |
| --- | --- | --- | --- | --- | --- | --- | --- | --- |
| 1 | quadratic | 100 | -2393661.47 | -2393649.85 | -2393601.16 |  |  |  |
| 2 | all quadratic | 1 : 69.5 2 : 30.5 % | -2067329.64 | -2067305.11 | -2067202.33 | 0.822 | 1: 9.8 2: 28.1 | 1: 0.96 2: 0.93 |
| 3 | all quadratic | 1 : 54.4 % 2 : 25.2 % 3 : 20.4 % | -2022501.04 | -2022463.6 | -2022306.72 | 0.739 | 1: 6.8 2: 16.2 3: 42.9 | 1: 0.89 2: 0.84 3: 0.92 |
| 4 | all quadratic | 1 : 54.4 % 2 : 21.4 % 3 : 13.9 % 4 : 10.3 % | -1992001.82 | -1991951.47 | -1991740.49 | 0.757 | 1: 7.7 2: 17.8 3: 24.0 4: 51.7 | 1: 0.90 2: 0.83 3: 0.79 4: 0.86 |
| 5 | all quadratic | 1 : 20.4 % 2 : 52.9 % 3 : 7.5 % 4 : 8.0 % 5 : 11.2 % | -1978468.84 | -1978405.58 | -1978140.5 | 0.754 | 1: 15.9 2: 7.4 3: 56.7 4: 37.1 5: 27.4 | 1: 0.80 2: 0.89 3: 0.82 4: 0.76 5: 0.77 |
| 6 | all quadratic | 1: 5.1 % 2: 52.3% 3: 17.6% 4: 8.4% 5: 8.9% 6: 7.7% | -1971334.46 | -1971258.29 | -1970939.11 | 0.743 | 1: 49.7 2: 6.6 3: 19.4 4: 37.0 5: 36.3 6: 50.6 | 1: 0.73 2: 0.88 3: 0.81 4: 0.77 5: 0.78 6: 0.81 |
| 7 | all quadratic ^†^ | 1: 6.4 % 2: 49.2% 3: 7.1% 4: 8.0% 5: 8.6% 6: 5.9% 7: 15.0% | -1961516.06 | -1961426.98 | -1961053.71 | 0.727 | 1: 32.8 2: 6.0 3: 49.7 4: 42.0 5: 39.6 6: 61.6 7: 18.9 | 1: 0.69 2: 0.85 3: 0.79 4: 0.78 5: 0.79 6: 0.79 7: 0.77 |
| 7 | outcome one: 2 2 2 2 2 2 2 outcome 2: 2 2 2 2 2 2 2 outcome 3: 0 2 2 2 2 2 2 ^X^ | 1: 5.2 % 2: 51.2% 3: 6.9% 4: 8.6% 5: 8.6% 6: 6.5% 7: 13.0% | -1957876.64 | -1957790.14 | -1957427.69 | 0.754 | 1: 48.5 2: 6.6 3: 42.0 4: 37.8 5: 43.8 6: 61.8 7: 20.9 | 1: 0.73 2: 0.87 3: 0.76 4: 0.78 5: 0.80 6: 0.81 7: 0.76 |
| 7* | outcome one: 2 2 2 2 2 2 2 outcome 2: 2 2 2 2 2 2 2 outcome 3: 0 2 2 1 2 2 2 | 1: 5.2 % 2: 51.2% 3: 6.9% 4: 8.6% 5: 8.6% 6: 6.5% 7: 13.0% | -1957868.98 | -1957783.77 | -1957426.73 | 0.754 | 1: 48.5 2: 6.6 3: 42.0 4: 37.8 5: 43.8 6: 61.8 7: 20.9 | 1: 0.73 2: 0.87 3: 0.76 4: 0.78 5: 0.80 6: 0.81 7: 0.76 |

Notes: BIC = Bayesian Information Criteria described for two different sample sizes: the total number of participants and the total number of observations., AIC = Akaike Information Criteria, OCC = odds of correct classification, AvePP = Average Posterior Probability. All models initially fitted with quadratic terms. Outcome 1 = GP contacts, Outcome 2 = psychotropic prescriptions, Outcome 3 = specialist contacts. ^†^ this model had a large standard error for Group 1, outcome 3 parameters with non-significant linear and quadratic terms, modelling was therefore repeated with an intercept-only model (0 order). ^X^ this model had a large standard error for group 4, outcome 3 so modelling was repeated with a linear term for this parameter. *** selected model.**

**Modelling processes**

Time was entered into the model as years since index presentation, each outcome was coded as a binary variable indicating present/absent within each complete year since index presentation (coded as 1(year 1), 2(year 2) etc. Because of the continuous nature of data collection within primary care records, time intervals were not subject to variance between cohort members. Trajectory shapes were refined by repeating the modelling procedure with lower order terms for model parameters where the standard error of the quadratic term was large.

**Supplementary table 6: cross-tabulation of region with group membership**

| Region | Group | | | | | | |  |
| --- | --- | --- | --- | --- | --- | --- | --- | --- |
|  | 1: low contact  n= 207,985 | 2: moderate non-pharmacological support  N= 43,836 | 3: declining contact, N=25,469 | 4: Year 4 escalating contact, N = 18,277 | 5: Year 5 escalating contact, N = 18,139 | 6: Prolonged GP contact, N= 32,147 | 7: Prolonged specialist contact,  N= 23,487 | Total |
|  | n  Column % | n  Column % | n  Column % | n  Column % | n  Column % | n  Column % | n  Column % | n  Column % |
| North East | 8,144 | 1,513 | 965 | 840 | 814 | 1392 | 750 | 14,418 |
|  | 3.9 | 3.5 | 3.8 | 4.6 | 4.5 | 4.3 | 3.2 | 3.9 |
| North West | 39,083 | 8,790 | 5,370 | 3,834 | 3,982 | 6,439 | 5,045 | 72,543 |
|  | 18.8 | 20.1 | 21.1 | 21.0 | 22.0 | 20.0 | 21.5 | 19.6 |
| Yorkshire And The Hum | 8,004 | 1,702 | 821 | 626 | 684 | 1,037 | 628 | 13,502 |
|  | 3.9 | 3.9 | 3.2 | 3.4 | 3.8 | 3.2 | 2.7 | 3.7 |
| East Midlands | 5,000 | 930 | 619 | 475 | 434 | 929 | 433 | 8,820 |
|  | 2.4 | 2.1 | 2.4 | 2.6 | 2.4 | 2.9 | 1.8 | 2.4 |
| West Midlands | 33,184 | 7,213 | 4,227 | 2,929 | 2,952 | 5,147 | 4,255 | 59,907 |
|  | 16.0 | 16.5 | 16.6 | 16.0 | 16.3 | 16.0 | 18.1 | 16.2 |
| East of England | 9,434 | 2,030 | 1,118 | 857 | 830 | 1,530 | 1,137 | 16,936 |
|  | 4.5 | 4.6 | 4.4 | 4.7 | 4.6 | 4.8 | 4.8 | 4.6 |
| South West | 26,522 | 6,077 | 3,212 | 2,448 | 2,431 | 4,199 | 2,109 | 46,998 |
|  | 12.8 | 13.9 | 12.6 | 13.4 | 13.4 | 13.1 | 9.0 | 12.7 |
| South Central | 26,614 | 5,665 | 3,753 | 2,422 | 2,390 | 4,444 | 3,476 | 48,764 |
|  | 12.8 | 12.9 | 14.7 | 13.3 | 13.2 | 13.8 | 14.8 | 13.2 |
| London | 32,902 | 6,062 | 2,867 | 2,077 | 1,984 | 3,488 | 3,341 | 52,721 |
|  | 15.8 | 13.8 | 11.3 | 11.4 | 10.9 | 10.9 | 14.2 | 14.3 |
| South East Coast | 18,857 | 3,767 | 2,500 | 1,760 | 1,620 | 3,524 | 2,273 | 34,301 |
|  | 9.1 | 8.6 | 9.8 | 9.6 | 8.9 | 11.0 | 9.7 | 9.3 |
| missing | 241 | 87 | 17 | 9 | 18 | 18 | 40 | 430 |
|  | 0.1 | 0.2 | 0.1 | 0.1 | 0.1 | 0.1 | 0.2 | 0.1 |

**Supplementary table 7: results of multinomial logistic regression analyses. Average probability of group membership by gender, ethnicity, IMD quintile and year of presentation**

| Baseline characteristic | *Group* |  |  |  |  |  |  |
| --- | --- | --- | --- | --- | --- | --- | --- |
|  | 1: low contact  N= 207,985 | 2:moderate non-pharmacological support N= 43,836 | 3: declining contact, N=25,469 | 4: Year 4 escalating contact,  N = 18,277 | 5: Year 5 escalating contact,  N = 18,139 | 6: Prolonged GP contact,  N= 32,147 | 7: Prolonged specialist contact,  N= 23,487 |
|  | Probability of group membership (% [95% CI]) | Probability of group membership (%[95% CI]) | Probability of group membership (%[95% CI]) | Probability of group membership (%[95% CI]) | Probability of group membership (%[95% CI]) | Probability of group membership (%[95% CI]) | Probability of group membership (%[95% CI]) |
|  | p | p | p | p | p | p | p |
| **Gender** | | | | | | | |
| *Female* | 56.6% [56.4 - 56.9] | 12.0% [11.8 - 12.2] | 4.9% [4.8 - 5.0] | 6.1% [6.0 - 6.2] | 5.7% [5.6 - 5.8] | 8.3% [8.2 - 8.5] | 6.3% [6.2 - 6.4] |
|  | <0.0005 | <0.0005 | <0.0005 | <0.0005 | <0.0005 | <0.0005 | <0.0005 |
| *Male* | 63.0% [62.8 - 63.3] | *11.4% [11.2 - 11.5]* | 4.6% [4.5 - 4.7] | 4.4% [4.3 - 4.5] | 4.4% [4.3 - 4.5] | 6.9% [6.8 - 7.0] | 5.2% [5.1 - 5.4] |
|  | *ref* | ref | ref | ref | ref | ref | ref |
| **Ethnicity** | | | | | | | |
| *White* | 57.0% [56.8 - 57.2%] | 12.0% [11.9% - 12.2%] | 5.1% [5.0% - 5.2%] | 5.7% [5.6% - 5.8%] | 5.4% [5.3% - 5.5%] | 8.5% [8.4% - 8.6%] | 6.3% [6.2% - 6.4%] |
|  | ref | ref | ref | ref | ref | ref | ref |
| *South Asian* | 69.7% [68.8% - 70.7%] | 10.1% [9.5% - 10.7%] | 4.1% [3.7% - 4.5%] | 3.0% [2.7% - 3.4%] | 3.8% [3.4% - 4.2%] | 4.7% [4.2% - 5.1%] | 4.6% [4.1% - 5.0%] |
|  | <0.0005 | <0.0005 | <0.0005 | <0.0005 | <0.0005 | <0.0005 | <0.0005 |
| *Black* | 69.3% [68.4% - 70.2%] | 11.7% [11.1% - 12.3%] | 3.3% [3.0% - 3.6%] | 3.5% [3.2% - 3.9%] | 3.4% [3.0% - 3.8%] | 4.1% [3.7% - 4.4%] | 4.6% [4.2% - 5.0%] |
|  | <0.0005 | 0.237 | <0.0005 | <0.0005 | <0.0005 | <0.0005 | <0.0005 |
| *Mixed* | 58.7% [57.6% - 59.8%] | 13.7% [13.0% - 14.4%] | 4.1% [3.7% - 4.5%] | 5.2% [4.7% - 5.7%] | 5.0% [4.5% - 5.5%] | 6.4%[5.9% - 6.9%] | 6.9% [6.3% - 7.5%] |
|  | 0.004 | <0.0005 | <0.0005 | 0.037 ^†^ | 0.2 | <0.0005 | 0.026 |
| *Other* | 67.7% [66.7% - 68.7%] | 11.2% [10.6% - 11.9%] | 3.5% [3.2% - 3.9%] | 3.7% [3.3% - 4.1%] | 3.8% [3.4% - 4.3%] | 4.7% [4.3% - 5.1%] | 5.2% [4.8% - 5.7%] |
|  | <0.0005 | 0.021 | <0.0005 | <0.0005 | <0.0005 | <0.0005 | <0.0005 |
| **IMD quintile** | | | | | | | |
| *1 (least deprived)* | 60.9% [60.5 - 61.3] | 11.6% [11.3 - 11.8] | 4.7% [4.6 - 4.9] | 5.0% [4.8 - 5.1] | 4.8% [4.6 - 4.9] | 7.4% [7.2 - 7.6] | 5.7% [5.5 - 5.8] |
|  | ref | ref | ref | ref | ref | ref | ref |
| *2* | 60.8% [60.4 - 61.1] | 11.9% [11.6 - 12.1] | 4.7% [4.6 - 4.9] | 5.1% [4.9 - 5.2] | 4.7% [4.5 - 4.9] | 7.3% [7.1 - 7.5] | 5.6% [5.4 - 5.7] |
|  | 0.577 | 0.104 | 0.775 | 0.359 | 0.627 | 0.673 | 0.376 |
| *3* | 59.8% [59.4 - 60.2] | 11.7% [11.4 - 11.9] | 4.8% [4.7 - 5.0] | 5.3% [5.1 - 5.5] | 5.0% [4.8 - 5.1] | 7.6% [7.4 - 7.8] | 5.8% [5.7 - 6.0] |
|  | <0.0005 | 0.593 | 0.441 | 0.008 | 0.103 | 0.126 | 0.181 |
| *4* | 59.9% [59.6 - 60.3] | 11.5% [11.2 - 11.7] | 4.7% [4.6 - 4.9] | 5.3% [5.1 - 5.4] | 5.1% [5.0 - 5.3] | 7.6% [7.5 - 7.8] | 5.9% [5.7 - 6.0] |
|  | <0.0005 | 0.485 | 0.805 | 0.015 | 0.002 | 0.066 | 0.130* |
| *5 (most deprived)* | 58.7% [58.4 - 59.0] | 11.9% [11.7 - 12.1] | 4.8% [4.7 - 5.0] | 5.5% [5.4 - 5.7] | 5.3% [5.2 - 5.5] | 7.9% [7.7 - 8.1] | 5.9% [5.7 - 6.0] |
|  | <0.0005 | 0.057* | 0.394 | <0.0005 | <0.0005 | <0.0005 | 0.117* |
| **Year of presentation** | | | | | | | |
| *2000 - 2004* | 66.1% [65.8 - 66.5] | 7.1% [6.9 - 7.3] | 6.2% [6.0 - 6.3] | 4.0% [3.9 - 4.2] | 4.7% [4.6 - 4.9] | 9.1% [8.9 - 9.3] | 2.7% [2.6 -2.8] |
|  | *ref* | ref | ref | ref | ref | ref | ref |
| *2005 to 2009* | 64.3% [64.0 - 64.6] | 10.1% [9.9 - 10.3] | 4.5% [4.4 - 4.7] | 4.8% [4.7 - 4.9] | 4.9% [4.8 - 5.0] | 7.3% [7.1 - 7.5] | 4.1% [4.0 - 4.2] |
|  | <0.0005 | <0.0005 | <0.0005 | <0.0005 | 0.097* | <0.0005 | <0.0005 |
| *2010 to 2016* | 55.3% [55.0 - 55.5] | 14.3% [14.1 - 14.5] | 4.3% [4.2 - 4.4] | 5.9% [5.8 - 6.0] | 5.2% [5.1 - 5.3] | 7.2% [7.0 - 7.3] | 7.8% [7.7 - 8.0] |
|  | <0.0005 | <0.0005 | <0.0005 | <0.0005 | <0.0005 | <0.0005 | <0.0005 |

Notes: Average probabilities of group memberships are derived from multinomial logistic regression models (one for each baseline characteristic) that adjust for age, type of mental health problem, and comorbidity using discrete marginal effects calculated with the margins command in Stata. P values indicate statistical significance of difference in average probability of group membership compared to reference category. Covariates (age, type of presentation and comorbidity) are held constant at average values for the cohort. *association significant in sensitivity analysis with weighting to account for probabilistic group membership assignment. † association not significant in sensitivity analysis with weighting to account for probabilistic group membership assignment

**Supplementary table 8: average probability of group membership by ethnicity for least and most deprived IMD quintile**

| *Ethnicity* | *IMD quintile* | 1: low contact  N= 207,985 | 2:moderate non-pharmacological support  N= 43,836 | 3: declining contact, N=25,469 | 4: Year 4 escalating contact,  N = 18,277 | 5: Year 5 escalating contact,  N = 18,139 | 6: Prolonged GP contact,  N= 32,147 | 7: Prolonged specialist contact,  N= 23,487 |
| --- | --- | --- | --- | --- | --- | --- | --- | --- |
|  |  | Average probability of group membership (%) | Probability of group membership (%) | Probability of group membership (%) | Probability of group membership (%) | Probability of group membership (%) | Probability of group membership (%) | Probability of group membership (%) |
| *White* | Least deprived | 58.3% [57.9 – 58.8] | 11.8% [11.5 – 12.1] | 5.1% [4.9 – 5.3] | 5.3% [5.1 – 5.5] | 5.1% [4.9 – 5.3] | 8.1% [7.9 - 8.4] | 6.2% [6.0 - 6.4] |
|  | Most deprived | 55.7% [55.3 – 56.0] | 12.3% [12.0 – 12.5] | 5.2% [5.0 – 5.3] | 6.0% [5.9 – 6.2] | 5.7% [5.6 - 5.9%] | 8.9% [8.7 - 9.1] | 6.3% [6.1 - 6.5] |
| *South Asian* | Least deprived | 71.2% [70.2 – 72.2] | 9.8% [9.1 – 10.4] | 4.0% [3.6 – 4.4] | 2.7% [2.4 – 3.1] | 3.5% [3.1 - 3.9%] | 4.4% [3.9 -4.8] | 4.4% [4.0 - 4.9] |
|  | Most deprived | 69.0% [68.0 – 70.0] | 10.3% [9.7 – 10.9] | 4.2% [3.8 – 4.6] | 3.1% [2.8 – 3.5] | 4.0% [3.6 -4.4%] | 4.9% [4.4 - 5.3] | 4.6% [4.1 - 5.0] |
| *Black* | Least deprived | 70.9% [70.0 – 71.9] | 11.3% [10.6 – 11.9] | 3.3% [2.9 – 3.6] | 3.2% [2.8 – 3.5] | 3.1% [2.7 - 3.5%] | 3.8% [3.4 - 4.1] | 4.5% [4.0 - 4.9] |
|  | Most deprived | 68.8% [67.8 – 69.7] | 11.9% [11.3 – 12.5] | 3.3% [3.0 – 3.7] | 3.7% [3.3 – 4.0] | 3.5% [3.2 - 3.9%] | 4.2% [3.8 - 4.6] | 4.6% [4.2 - 5.0] |
| *Mixed* | Least deprived | 60.2% [59.0 – 61.3] | 13.4% [12.7 – 14.2] | 4.1% [3.7 – 4.5] | 4.8% [4.3 – 5.3] | 4.7% [4.2 - 5.2%] | 6.0% [5.5 - 6.6] | 6.8% [6.2 - 7.4] |
|  | Most deprived | 57.7% [56.5 – 58.8] | 14.0% [13.2 – 14.7] | 4.2% [3.7 – 4.6] | 5.4% [4.9 – 5.9] | 5.3% [4.8 - 5.8%] | 6.6% [6.0 - 7.1] | 6.9% [6.3 - 7.5] |
| *Other* | Least deprived | 69.0% [68.0 – 70.1] | 11.0% [10.3 – 11.6] | 3.5% [3.1 – 3.9] | 3.3% [3.0 – 3.8] | 3.6% [3.2 - 4.0%] | 4.4% [4.0 - 4.9] | 5.1% [4.6 - 5.6] |
|  | Most deprived | 66.8% [65.7 – 67.8] | 11.5% [10.8 – 12.2] | 3.6% [3.2 – 4.0] | 3.9% [3.4 – 4.3] | 4.1% [3.6 - 4.5%] | 4.9% [4.4 - 5.4] | 5.3% [4.8 - 5.7] |

Notes: average predicted probabilities were derived using stata’s margins command following multinomial logistic regression adjusted for IMD quintile, age, mental health/neurodevelopmental category and comorbidity as covariates. Covariates (age, type of presentation and comorbidity) are held constant at average values.

**Supplementary table 9: probability of group membership by baseline clinical characteristics**

| **Mental health category at baseline** | | | | | | | | |  |
| --- | --- | --- | --- | --- | --- | --- | --- | --- | --- |
|  | 1: low contact  N= 207,985 | 2:moderate non-pharmacological support N= 43,836 | 3: declining contact, N=25,469 | 4: Year 4 escalating contact,  N = 18,277 | 5: Year 5 escalating contact,  N = 18,139 | 6: Prolonged GP contact,  N= 32,147 | 7: Prolonged specialist contact,  N= 23,487 | Total | |
|  | n  Row % | n  Row % | n  Row % | n  Row % | n  Row % | n  Row % | n  Row % | n  Row % | |
| ASD | 20,082 | 4,685 | 900 | 1,328 | 995 | 2,839 | 1,265 | 32,094 | |
|  | 62.6 | 14.6 | 2.8 | 4.1 | 3.1 | 8.9 | 3.9 | 100 | |
| ADHD | 9,480 | 2,467 | 2,221 | 1,240 | 797 | 6,962 | 3,112 | 26,279 | |
|  | 36.1 | 9.4 | 8.5 | 4.7 | 3.0 | 26.5 | 11.8 | 100 | |
| psychosis | 1,674 | 464 | 324 | 174 | 198 | 410 | 1,032 | 4,651 | |
|  | 39.2 | 10.9 | 7.6 | 4.1 | 4.6 | 9.6 | 24.1 | 100 | |
| depression | 42,469 | 6,111 | 12,863 | 5,192 | 6,504 | 11,427 | 8,548 | 100,826 | |
|  | 45.6 | 6.6 | 13.8 | 5.6 | 7.0 | 12.3 | 9.2 | 100 | |
| anxiety | 49,752 | 8,419 | 6,659 | 4,520 | 4,892 | 6,730 | 5,624 | 91,627 | |
|  | 57.5 | 9.7 | 7.7 | 5.2 | 5.7 | 7.8 | 6.5 | 100 | |
| conduct | 2,588 | 703 | 214 | 163 | 163 | 309 | 203 | 4,622 | |
|  | 59.6 | 16.2 | 4.9 | 3.8 | 3.8 | 7.1 | 4.7 | 100 | |
| self-harm | 16,361 | 3,186 | 2,399 | 2,211 | 1,912 | 1,774 | 2,469 | 33,569 | |
|  | 54.0 | 10.5 | 7.9 | 7.3 | 6.3 | 5.9 | 8.2 | 100 | |
| eating disorder | 9,615 | 1,452 | 701 | 532 | 536 | 624 | 824 | 15,211 | |
|  | 67.3 | 10.2 | 4.9 | 3.7 | 3.8 | 4.4 | 5.8 | 100 | |
| Tics, childhood-specific disorders | 15,182 | 2,994 | 571 | 641 | 664 | 901 | 598 | 22,774 | |
|  | 70.5 | 13.9 | 2.7 | 3.0 | 3.1 | 4.2 | 2.8 | 100 | |
| Behavioural | 48,083 | 15,246 | 1,536 | 3,216 | 2,698 | 3,438 | 2,853 | 82,295 | |
|  | 62.4 | 19.8 | 2.0 | 4.2 | 3.5 | 4.5 | 3.7 | 100 | |
| Comorbid | 6,947 | 1,768 | 2,750 | 907 | 1,144 | 3,092 | 2,805 | 19,413 | |
|  | 35.8 | 9.1 | 14.2 | 4.7 | 5.9 | 15.9 | 14.4 | 100 | |

**Supplementary figure 3: sensitivity analysis**


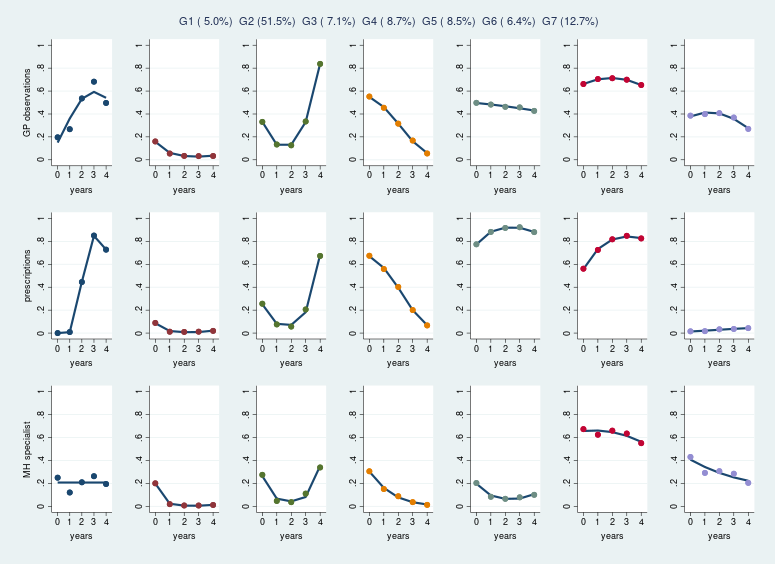


Notes: group-based trajectory modelling was repeated with only cohort members followed up for >= 5 years. Weighting was used to account for non-random attrition over time as we expected that attrition would be associated with baseline characteristics. The weighting variable was the inverse of the probability of a participant completing at least 5 years of follow-up from a logistic regression model with all baseline characteristics as independent variables, and completion of >= 5 years follow-up as the dependent variable. Group numbering differs from the trajectory model in main analysis, but colour-coding is the same. BIC=-1690473.47 (N_observations_=4279005), BIC=-1690384.10 (N_participants_=285267), AIC=-1690035.58

**GRoLTS checklist**^13^

|  | Checklist Item | Reported? |
| --- | --- | --- |
| 1 | Is the metric of time used in the statistical model reported? | Yes p6, appendix p18 |
| 2 | Is information presented about the mean and variance of time within a wave? | N/A, appendix p18 |
| 3a. | Is the missing data mechanism reported? | Yes p7 |
| 3b. | Is a description provided of what variables are related to attrition/missing data? | Yes appendix p7 |
| 3c. | Is a description provided of how missing data in the analyses were dealt with? | yes p7 |
| 4 | Is information about the distribution of the observed variables included? | Yes p6 |
| 5 | Is the software mentioned? | Yes p7 |
| 6a. | Are alternative specifications of within-class heterogeneity considered (e.g., LGCA vs. LGMM) and clearly documented? If not, was sufficient justification provided as to eliminate certain specifications from consideration? | not applicable to GBMTM |
| 6b. | Are alternative specifications of the between-class differences in variance covariance matrix structure considered and clearly documented? If not, was sufficient justification provided as to eliminate certain specifications from consideration? | not applicable to GBMTM |
| 7 | Are alternative shape/functional forms of the trajectories described? | Yes appendix p18 |
| 8 | If covariates have been used, can analyses still be replicated? | Not used |
| 9 | Is information reported about the number of random start values and final iterations included? | N/A |
| 10 | Are the model comparison (and selection) tools described from a statistical perspective? | Yes p7 |
| 11 | Are the total number of fitted models reported, including a one-class solution? | Yes appendix p17-18 |
| 12 | Are the number of cases per class reported for each model (absolute sample size, or proportion)? | Yes appendix p17-18 |
| 13 | If classification of cases in a trajectory is the goal, is entropy reported? | Yes appendix p17-18 |
| 14a. | Is a plot included with the estimated mean trajectories of the final solution? | Yes figure 3 |
| 14b. | Are plots included with the estimated mean trajectories for each model? | No - available on request |
| 14c. | Is a plot included of the combination of estimated means of the final model and the observed individual trajectories split out for each latent class? | No |
| 15 | Are characteristics of the final class solution numerically described (i.e., means, SD/SE, n, CI, etc.)? | Yes p8/figure 3 |
| 16 | Are the syntax files available (either in the appendix, supplementary materials, or from the authors)? | Yes p7 |

References

1 Mathur R, Bhaskaran K, Chaturvedi N, *et al.* Completeness and usability of ethnicity data in UK-based primary care and hospital databases. *J Public Health (Oxf)* 2014; **36**: 684–92.

2 English indices of deprivation 2015 - GOV.UK. https://www.gov.uk/government/statistics/english-indices-of-deprivation-2015 (accessed April 3, 2023).

3 PERSON GENDER CODE. https://www.datadictionary.nhs.uk/attributes/person_gender_code.html (accessed March 13, 2023).

4 Kendrick T, Stuart B, Newell C, Geraghty AWA, Moore M. Changes in rates of recorded depression in English primary care 2003-2013: Time trend analyses of effects of the economic recession, and the GP contract quality outcomes framework (QOF). *J Affect Disord* 2015; **180**: 68–78.

5 John A, McGregor J, Fone D, *et al.* Case-finding for common mental disorders of anxiety and depression in primary care: an external validation of routinely collected data. *BMC Med Inform Decis Mak* 2016; **16**: 35.

6 Cybulski L, Ashcroft DM, Carr MJ, *et al.* Temporal trends in annual incidence rates for psychiatric disorders and self-harm among children and adolescents in the UK, 2003–2018. *BMC Psychiatry* 2021; **21**: 229.

7 Hagberg KW, Jick SS. Validation of autism spectrum disorder diagnoses recorded in the Clinical Practice Research Datalink, 1990–2014. *Clin Epidemiol* 2017; **9**: 475.

8 Houghton R, Liu C, Bolognani F. Psychiatric Comorbidities and Psychotropic Medication Use in Autism: A Matched Cohort Study with ADHD and General Population Comparator Groups in the United Kingdom. *Autism Res* 2018; **11**: 1690–700.

9 Abel KM, Hope H, Swift E, *et al.* Prevalence of maternal mental illness among children and adolescents in the UK between 2005 and 2017: a national retrospective cohort analysis. *Lancet Public Heal* 2019; **4**: e291–300.

10 Taylor D, Barnes TE, Young A. The Maudsley prescribing guidelines in psychiatry, 13th editi. Hoboken, NJ: Wiley Blackwell, 2018.

11 Overview | Attention deficit hyperactivity disorder: diagnosis and management | Guidance | NICE. https://www.nice.org.uk/guidance/ng87 (accessed April 3, 2023).

12 Newlove-Delgado T, Ford TJ, Hamilton W, Stein K, Ukoumunne OC. Prescribing of medication for attention deficit hyperactivity disorder among young people in the Clinical Practice Research Datalink 2005–2013: analysis of time to cessation. *Eur Child Adolesc Psychiatry* 2018; **27**: 29.

13 van de Schoot R, Sijbrandij M, Winter SD, Depaoli S, Vermunt JK. The GRoLTS-Checklist: Guidelines for Reporting on Latent Trajectory Studies. *Struct Equ Model A Multidiscip J* 2017; **24**: 451–67.
